# Electrified dry reforming of methane on Ni-La_2_O_3_–loaded activated carbon: A net CO_2_-negative reaction

**DOI:** 10.1126/sciadv.adv1585

**Published:** 2025-07-11

**Authors:** Wei Zhao, Xueyi Mei, Yexin Zhang, Zhenghui Zhang, Kai Chen, Weiping Xie, Ying Xin, Zhaoliang Zhang, Jian Zhang

**Affiliations:** ^1^ Ningbo Institute of Materials Technology and Engineering, Chinese Academy of Sciences, 1219 Zhongguan West Road, Ningbo 315201, People’s Republic of China.; ^2^ School of Chemistry and Chemical Engineering, University of Jinan, 336 West Road of Nan Xinzhuang, Jinan 250022, People’s Republic of China.; ^3^ University of the Chinese Academy of Sciences, 19A Yuquan Road, Beijing 100049, People’s Republic of China.; ^4^ Institute of Catalysis for Energy and Environment, College of Chemistry and Chemical Engineering, Shenyang Normal University, Shenyang, 110034 Liaoning, People’s Republic of China.

## Abstract

Dry reforming of methane (DRM) offers an attractive solution for the high-value utilization of two primary greenhouse gasses, methane and carbon dioxide, but its industrial applications are hindered by the net carbon dioxide–positive emissions resulting from substantial energy consumption. Here, we developed an electrified technology by passing electric current through a conductive catalyst constituting nickel and lanthanum(III) oxide cosupported on activated carbons, reaching thermodynamic equilibrium conversions and sustaining for at least 120 hours. Notably, the energy efficiency (2.976 millimoles per kilojoule) exceeds all the previously reported values, enabling net carbon dioxide–negative emissions powered by common sustainable electricity. An electrically driven release of lattice oxygen bridges the decomposition of the lanthanum oxycarbonate intermediate generated from carbon dioxide activation and the reduction of the resultant nickel oxide species by deposited carbon from methane dissociation, thus inhibiting excessive nickel oxidation and lanthanum(III) oxide carbonatation. The electrified approach would make DRM a true pathway mitigating overall carbon footprint and combating global warming.

## Teaser

Electrification cuts the energy cost for dry (CO_2_) reforming of CH_4_, enabling net CO_2_-negative emissions with low-carbon powers.

## INTRODUCTION

Carbon dioxide (CO_2_) and methane (CH_4_) are two of the most important greenhouse gasses, with their global emissions reaching 37.1 and 10.5 billion tons of CO_2_ equivalent in 2022, accounting for 75 and 20% of overall contributions to global warming, respectively (https://ourworldindata.org/greenhouse-gas-emissions). Dry reforming of methane (DRM), specifically the reaction between CH_4_ and CO_2_ to produce syngas with a H_2_/CO molar ratio of unity according to Eq. 1, holds immense promise for simultaneously converting them into valuable chemical products with a circular carbon economy (*1*–*5*). The syngas can be used for direct dimethyl ether synthesis, methanol production, or the Fischer-Tropsch synthesis of long-chain hydrocarbons or aromatics (*6*–*9*). In particular, DRM enables the direct utilization of CO_2_-rich natural gas and biogas produced by anaerobic digestion as the feedstock (*9*, *10*)

$${\text{CH}}_{4}+{\text{CO}}_{2}\to \text{2CO}+{\text{2H}}_{2}\;{\text{∆H}}_{29\text{8K}}=247\;\text{kJ}\;{{\text{mol}}_{\text{CO2}}}^{-1}$$ (1)

Despite great efforts made in the laboratory with the expectation of CO_2_ utilization, DRM has not yet been implemented in industrial settings. One of the most important reasons is the massive CO_2_ emission that far exceeds CO_2_ conversion achieved in practical applications, which stems from its strongly endothermic nature with high reaction enthalpy, necessitating extremely high working temperatures (>800°C) to attain high equilibrium conversion to syngas (*11*). Moreover, the intense heat supplied to DRM is typically sourced from the combustion of fossil fuels, consequently resulting in net CO_2_-positive emissions, which contradicts the goal of CO_2_ emission relief (*8*, *12*).

To mitigate global CO_2_ emissions in chemical productions, there is an emerging growth of low-carbon electricity such as solar and wind energies being directly used to drive chemical reactions, replacing the reliance on fossil fuels (*12*–*17*). For instance, Wismann *et al.* (*18*) proposed an electrified steam-CH_4_ reforming based on Joule heating of FeCrAl alloy tube coated with Ni catalysts, realizing the reaction to thermal equilibrium and expecting to reduce nearly 1% of all CO_2_ emission if used globally. A similar electrification strategy has also been explored for DRM, with the Joule heating of FeCrAl alloy (*19*), SiSiC foams (*20*, *21*), or carbon fiber paper (*22*). In particular, Yu *et al.* (*22*) revealed that the high peak temperatures (>900°C) induced by pulsed Joule heating facilitate the in situ regeneration of catalysts from coking during DRM.

The above Joule heating of conductive monolithic supports is used only to ameliorate the way of heating to enhance performance and energy efficiency. Tour and colleagues (*23*) disclosed that passing electric current through conductive materials involves not only a thermal effect but also an electrical effect under electrical potentials, which would inherently lower the activation energy of reactions and accelerate chemical processes. Recently, we used conductive materials such as antimony-tin oxide (ATO) and LaCoO_3_ perovskite for electrified soot combustion and NO*_x_* storage-reduction, demonstrating substantial activity enhancement (*24*–*28*). Electrically driven lattice oxygen participates in reactions and is responsible for an unprecedented decrease in the ignition temperatures of diesel soot (*24*). Adopting a similar catalytic system, Zheng *et al.* (*29*) found that electrical current weakened V─O bonds on V_1_/ATO for selective catalytic reduction of NO*_x_* with NH_3_, thereby achieving complete NO conversion to N_2_ at ultralow temperatures and V contents. Upon reviewing the DRM mechanisms, lattice oxygen is also involved in the oxidation of the deposited carbon from CH_4_ dissociation (*11*, *30*–*32*). Therefore, the electrification of DRM using conductive catalysts holds great promise to promote the decomposition of oxygen-containing intermediates and subsequent oxidation of coke and to simultaneously decrease energy consumption.

Here, we report an electrified DRM (e-DRM) reaction based on conductive catalysts, reaching thermodynamic equilibrium conversions and attaining net CO_2_-negative emissions when powered by low-carbon electricity. The conductive catalyst was fabricated by cosupporting metallic Ni and La_2_O_3_ on activated carbon (Ni-La_2_O_3_/AC), as Ni and La_2_O_3_ have been generally used as the active component and promoter for catalytic DRM, respectively (*33*, *34*), while Ni and AC offer the catalyst with electrical conductivity. The e-DRM was actuated by applying electric power to the conductive catalyst, demonstrating thermodynamic equilibrium conversions and sustaining for at least 120 hours. In stark contrast, the traditional thermal DRM (t-DRM) shows inferior activity and deactivates within 10 hours. In particular, the energy efficiency has been raised to a record-high level compared to the previously reported results, thereby enabling the utilization of low-carbon electricity to achieve net CO_2_-negative emissions. Mechanism studies verified that it is the electrically driven lattice oxygen that accelerates the DRM process via Ni and La_2_O_3_ synergy. The e-DRM on conductive catalysts may pave the way for its industrialization featuring the simultaneous meeting of the environmental, social, and governance criteria.

## RESULTS

### Electrified reaction system and performance evaluation

**Fig. 1.**
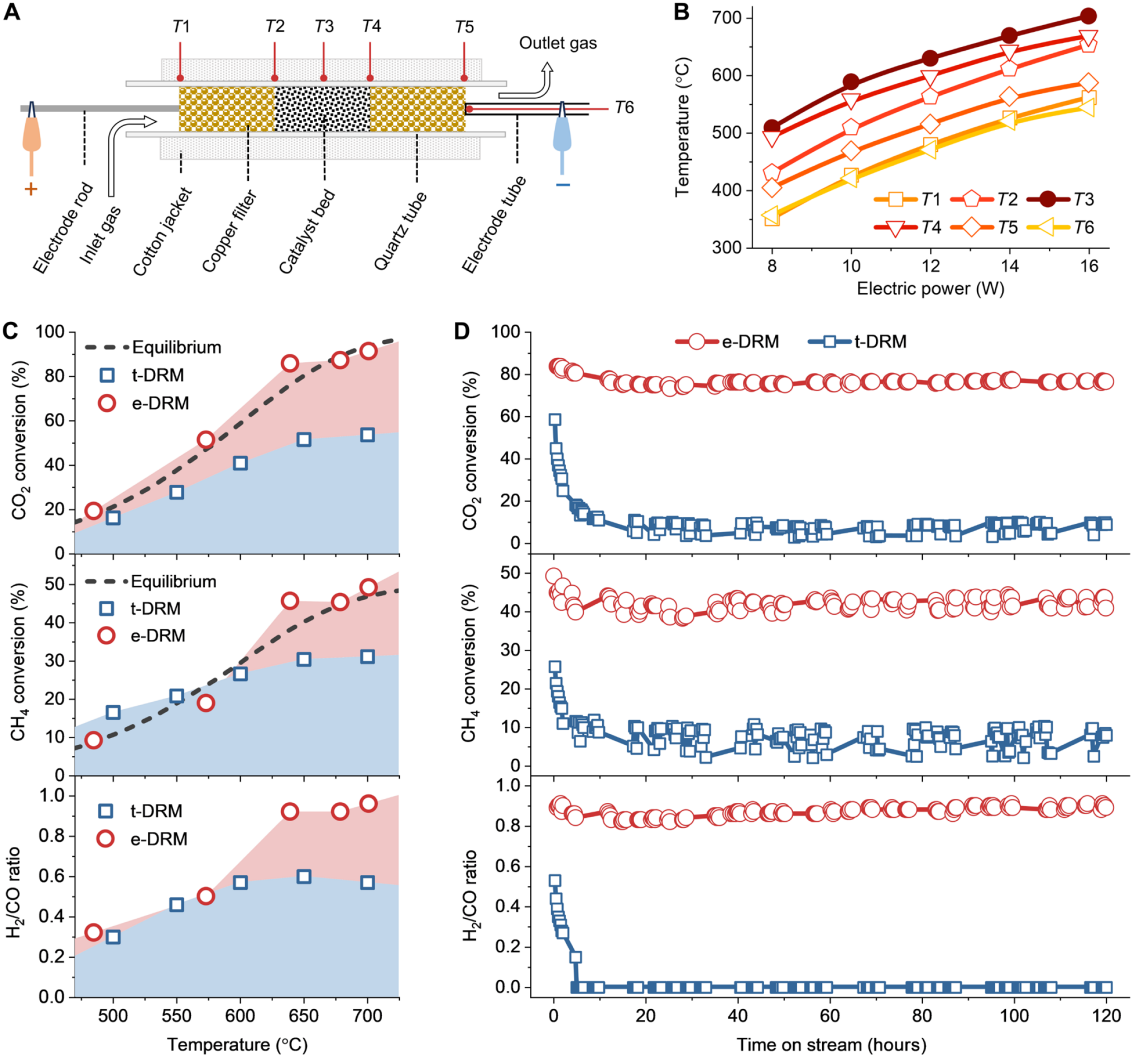
Assembly of the homemade reactor for e-DRM and performance comparison. (**A**) Scheme of the homemade reactor for e-DRM and arrangement of six thermocouples on the reactor labeled as *T*1 to *T*6. (**B**) Temperatures measured by thermocouples *T*1 to *T*6 under different electric powers for e-DRM. (**C**) Performance comparison between e-DRM and t-DRM in CO_2_ and CH_4_ conversions and H_2_/CO ratio. The thermodynamic equilibriums for CO_2_ and CH_4_ conversions are plotted as dashed lines. (**D**) Comparison of catalyst durability between the e-DRM and the t-DRM along time on stream. The e-DRM was conducted at 16 W, reaching around 700°C, whereas the t-DRM lasted for 10 hours at 700°C. All reactions were conducted over Ni-La_2_O_3_/AC with a CH_4_/CO_2_ ratio of 2 and a GHSV of 21 liters hour^−1^ g_cat_^−1^. The Ni-La_2_O_3_/AC catalyst has a Ni content of 4 wt % and a La/Ni atomic ratio of 2.

For a typical e-DRM, 0.2 g of Ni-La_2_O_3_/AC catalyst was packed into a homemade reactor. As depicted in Fig. 1A, the catalyst was sandwiched between two copper filters within a quartz tube, and the assembly was connected to an adjustable DC power, forming a circuit. The reactor construction allows electric current to pass through the catalyst bed in conjunction with the flowing reaction gas. A cotton jacket was used to wrap the reactor for heat preservation. When electric power is applied, the electrothermal effect leads to a temperature increase in the catalyst bed. To get accurate temperatures, six thermocouples were positioned at different points of the reactor as schemed in Fig. 1A (*T*1 to *T*6). Among them, the temperature at the center of the reactor tube’s outside surface (*T*3) is higher than that of other points (Fig. 1B). Given that the evaluated e-DRM performance as a function of the highest temperature precisely corresponds with the thermodynamic equilibrium of DRM (discussed later), the *T*3 temperature can be considered as the temperature of the catalyst bed. The *T*4 temperature, which corresponds to the measurement location near the catalyst bed exit (Fig. 1A), is slightly lower than the *T*3 temperature by <35°C (Fig. 1B), indicating that the temperature gradient within the catalyst bed is not distinctive. When the electric power input is elevated to 16 W, with the voltages below 13 V and the current below 2 A (fig. S1), the catalyst temperature rises to 700°C (Fig. 1B). Furthermore, the gas hourly space velocity (GHSV) has little influence on the catalyst temperature (fig. S2), suggesting that the heat transfer from the catalyst to the reaction gas can be neglected.

To achieve a stable and optimal performance of e-DRM, we first studied the composition of Ni-La_2_O_3_/AC catalyst and reaction conditions. A Ni content of 4 wt % (fig. S3) and La/Ni atomic ratio of 2 (fig. S4) were singled out, while the GHSV was determined to be 21 liters hour^−1^ g_cat_^−1^ to minimize the external diffusion resistance of e-DRM (fig. S5) (*35*, *36*). In addition, the catalyst particle size and the corresponding resistance of the catalyst bed exhibit minimal influence on the e-DRM performance (fig. S6). The feed molecular ratio of CH_4_/CO_2_ was selected as 2, taking into account the following considerations: (i) When the ratio is below 2, the AC support is apt to be gasified by CO_2_, i.e., reverse Boudouard reaction (C + CO_2_ → 2CO) (fig. S7) (*37*–*39*), which can be suppressed by the increase in CH_4_ feeding (*4*); (ii) the CH_4_/CO_2_ ratio of 2 is similar to the composition of raw biogas, which consists of 60 to 65% CH_4_ and 30 to 35% CO_2_ (*6*, *40*).

Using the above optimized Ni-La_2_O_3_/AC catalyst and reaction conditions, the e-DRM was compared with the t-DRM within a temperature range of 500° to 700°C. As plotted in Fig. 1C, the e-DRM outperforms the t-DRM in CO_2_ and CH_4_ conversions and H_2_/CO ratio at above 600°C. The t-DRM performance is similar to that of the Ni/La_2_O_3_ catalyst without AC supports but with the same Ni content (4 wt %) (fig. S8). The performance improvement of the e-DRM compared to the t-DRM presumably originates from the contribution of the electrical effect (*23*, *29*), which can be quantified using their performance differences (red areas in Fig. 1C). Furthermore, the electrical effect is augmented with the increasing temperature (electric power), as demonstrated by the fact that the CO_2_ and CH_4_ conversions of the e-DRM are nearly at thermodynamic equilibriums (Fig. 1C and fig. S9), while those of the t-DRM decelerate with the increasing temperature.

The H_2_/CO ratios of the e-DRM are closer to 1 above 600°C compared to those of the t-DRM (Fig. 1C), indicating the reverse water-gas shift (RWGS) reaction (CO_2_ + H_2_ → CO + H_2_O) is suppressed, while another secondary reaction (AC gasification) is progressively inhibited, as evidenced by isotopic e-DRM experiments using ^13^CH_4_ and ^13^CO_2_ feeds, which show a decline in ^12^CO generation over time (fig. S10). Furthermore, the e-DRM exhibits superior durability to the t-DRM in a test for 120 hours at 700°C (Fig. 1D). In contrast with the sharp drop of the t-DRM performance within 10 hours, the e-DRM performance only experiences a slight decrease during the initial 10 hours and then maintains a CO_2_ conversion of 77% and a H_2_/CO ratio of 0.9 for the subsequent 110 hours.

### Energy efficiency and CO_2_ emission for e-DRM

**Fig. 2.**
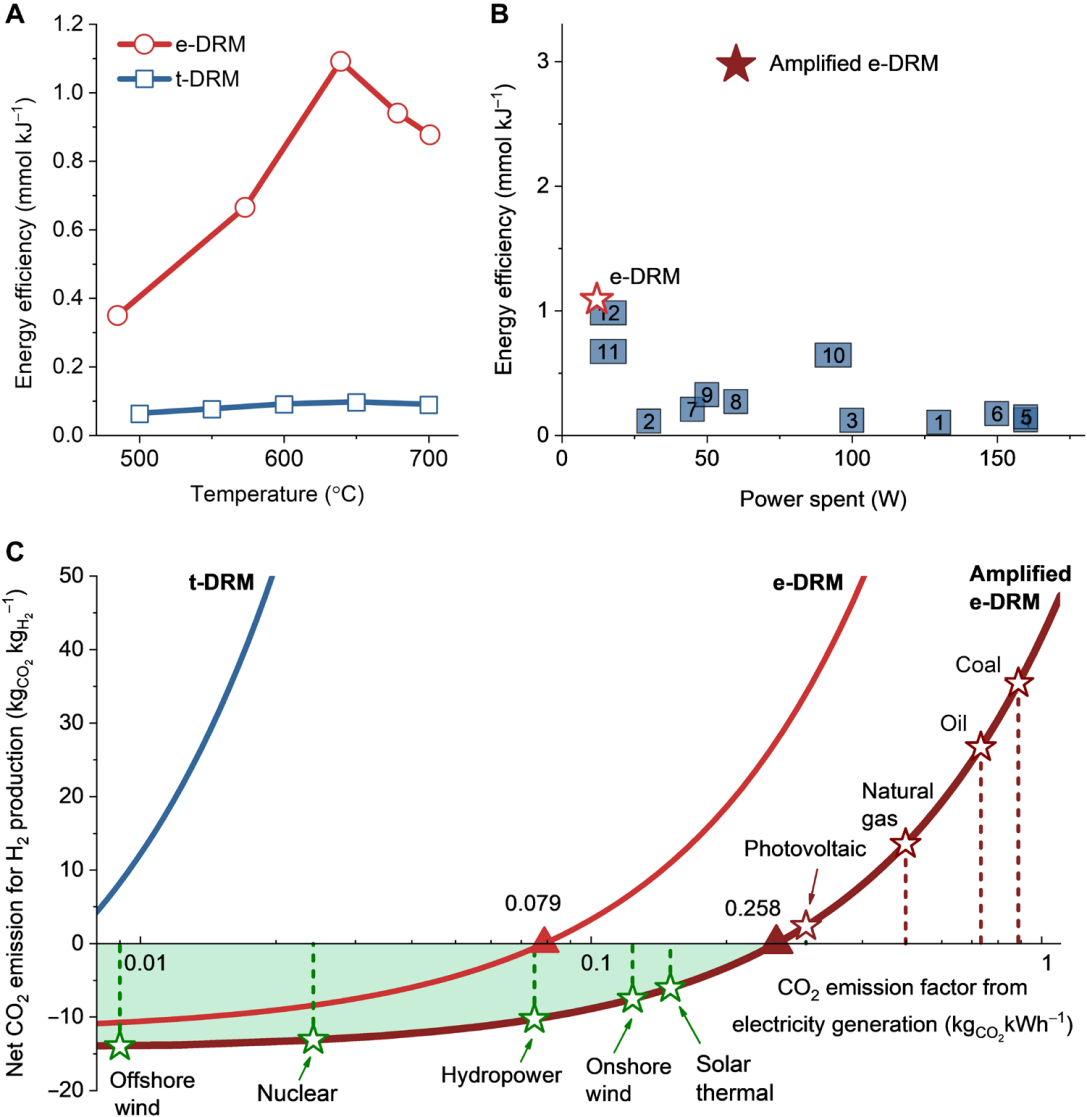
Energy efficiency of e-DRM. (**A**) Comparison of energy efficiency between the e-DRM and the t-DRM. Both reactions were conducted over Ni-La_2_O_3_/AC with a CH_4_/CO_2_ ratio of 2 and a GHSV of 21 liters hour^−1^ g_cat_^−1^. The catalyst has a Ni content of 4 wt % and a La/Ni atomic ratio of 2. (**B**) Comparison of energy efficiency between the e-DRM and the amplified e-DRM and the previously reported DRM results labeled by the entries in table S1. (**C**) Plots of net CO_2_ emission for H_2_ production in DRM against CO_2_ emission factor from electricity generation. The data of e-DRM come from the reaction with 0.2 g of Ni-La_2_O_3_/AC catalyst at 12 W, whereas the data of amplified e-DRM originate from the reaction with 5.4 g of the same catalyst at 60 W. The amplified e-DRM maintains the same GHSVs for CH_4_ (5.6 liters hour^−1^ g_cat_^−1^) and CO_2_ (2.8 liters hour^−1^ g_cat_^−1^) flows.

The electrification approach is anticipated to reduce the energy consumption of DRM, contributing to a decrease in net CO_2_ emission. The energy efficiency was quantified by considering the total number of molecules of converted CH_4_ and CO_2_ per 1 kJ of energy consumed, which is one of the pivotal indicators for different DRM catalytic systems (*41*). As shown in Fig. 2A, the energy efficiency of e-DRM greatly exceeds that of t-DRM at the same temperatures, by a factor of 6 to 12 times. The highest energy efficiency of e-DRM is 1.09 mmol kJ^−1^ with an electrical power of 12 W. This surpasses all the reported values for extensively studied plasma-catalytic DRM (the data points labeled 1 to 11 in Fig. 2B) (*42*–*52*) and an emerging pulsed laser-catalytic DRM (the data point labeled 12 in Fig. 2B) (*41*).

**Fig. 3.**
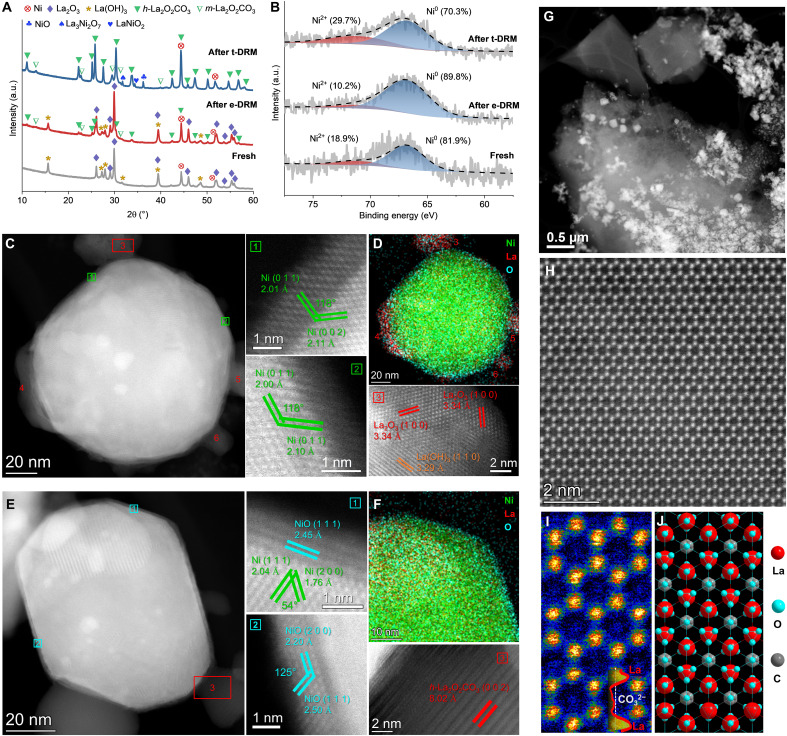
Identification of DRM intermediates. (**A**) X-ray diffraction (XRD) patterns of the fresh Ni-La_2_O_3_/AC catalyst and the spent catalysts after the e-DRM and the t-DRM. The diffractions of Ni (JCPDS 00-004-0850), La_2_O_3_ (JCPDS 00-005-0602), La(OH)_3_ (JCPDS 01-083-2034), hexagonal La_2_O_2_CO_3_ (*h-*La_2_O_2_CO_3_; JCPDS 04-009-3944), monoclinic La_2_O_2_CO_3_ (*m-*La_2_O_2_CO_3_; JCPDS 00-048-1113), NiO (JCPDS 01-071-4750), La_3_Ni_2_O_7_ (JCPDS 00-050-0244), and LaNiO_2_ (JCPDS 04-011-8132) are marked. a.u., arbitrary units. (**B**) Ni 3p x-ray photoelectron spectroscopy (XPS) spectra of the fresh Ni-La_2_O_3_/AC catalyst and the spent catalysts after the e-DRM and the t-DRM. (**C** and **D**) High-angle annular dark field–scanning transmission electron microscopy (HAADF-STEM) analysis on the individual Ni particles of the Ni-La_2_O_3_/AC catalysts spent after the e-DRM (C) and accompanying energy-dispersive x-ray (EDX) mapping (D). The lattice analysis on the areas numbered 1 to 3 is separately shown, and the areas numbered 3 to 6 represent La_2_O_3_/La(OH)_3_ substrates. (**E** and **F**) HAADF-STEM analysis on the individual Ni particles of the Ni-La_2_O_3_/AC catalysts spent after the t-DRM (E) and accompanying EDX mapping (F). The lattice analysis on the areas numbered 1 to 3 is separately shown. (**G** to **I**) HAADF-STEM images of the La_2_O_2_CO_3_ intermediate appeared on the Ni-La_2_O_3_/AC catalysts spent after the t-DRM. A corresponding HAADF intensity line scanning is shown in the subfigure of (I). (**J**) Crystal structure of *h*-La_2_O_2_CO_3_ projected onto the (0 0 4) lattice plane. The e-DRM was conducted at 16 W for 10 hours, reaching around 700°C, whereas the t-DRM lasted for 10 hours at 700°C. All reactions were conducted over Ni-La_2_O_3_/AC with a CH_4_/CO_2_ ratio of 2 and a GHSV of 21 liters hour^−1^ g_cat_^−1^. The Ni-La_2_O_3_/AC catalyst has a Ni content of 4 wt % and a La/Ni atomic ratio of 2.

Considering that the utilization of electric power for e-DRM is not sufficient with a small reaction scale due to a large proportion of energy dissipation, an amplified e-DRM was conducted, in which the Ni-La_2_O_3_/AC catalyst was augmented from 0.2 to 5.4 g, while the GHSVs for CH_4_ (5.6 liters hour^−1^ g_cat_^−1^) and CO_2_ (2.8 liters hour^−1^ g_cat_^−1^) flows were maintained. Although scale-up effects reduce the DRM efficiency to some extent, the energy efficiency is enlarged to a range of 2.5 to 3 mmol kJ^−1^, with the power spent at 45 to 70 W (fig. S11). When the electric power is 60 W, the highest energy efficiency achieved is 2.976 mmol kJ^−1^, representing a threefold increase compared to that of the unamplified one (Fig. 3B). Furthermore, a dimensionless energy efficiency was also calculated (note S1), which is the ratio of the overall enthalpy gain across the reactor to the electrical energy input (*20*). After the amplification, this efficiency substantially rises from 17 to 80%, implying that only 20% of the electrical energy input is lost in the amplified e-DRM.

The CO_2_ emissions from the generation of electricity spent offset the CO_2_ consumption of DRM itself. The net CO_2_ emission based on H_2_ production (*C*_H2_) for DRM is calculated by the following equation

$$C_{\text{H2}}=E_{\text{H2}}\times \mu -W_{\text{CO}2/H2}$$ (2)

where *E*_H2_ and *W*_CO2/H2_ represent the energy consumption and the CO_2_ consumption for H_2_ production from DRM, while μ is the CO_2_ emission factor for electricity generation, which is available from life cycle assessments (LCAs) (*53*).

The calculated net CO_2_ emission for H_2_ production is plotted against the CO_2_ emission factor (μ) for electricity generation in Fig. 2C and also listed in table S2. The t-DRM always produces positive CO_2_ emission even when powered by sustainable electricity, whereas the e-DRM demonstrates substantially reduced CO_2_ emission. In particular, for the e-DRM, the net CO_2_ emission becomes negative when the CO_2_ emission from electricity generation is below 0.079 kg_CO2_ kWh^−1^, which includes the electricity generated from offshore wind, nuclear sources, and hydropower. Regarding the amplified e-DRM, the net CO_2_ emission becomes even more negative, and the threshold of CO_2_ emission factor for electricity generation to achieve net CO_2_-negative emission increases to 0.258 kg_CO2_ kWh^−1^. Consequently, the electricity sources are expanded to a larger scope, offshore wind, nuclear, hydropower, onshore wind, and solar thermal. In addition, the use of photovoltaic electricity also enables a near-zero emission. We believe that there is still a large room for improvement on energy efficiency and negative emissions when the e-DRM continues to be scaled up to pilot or industry levels. Although LCAs for the e-DRM have not been done because of the insufficiency of data, the achievement of net CO_2_-negative emissions during the reaction section provides a prerequisite for attaining overall negative or near-zero CO_2_ emissions (*12*).

### Identification of DRM intermediates

The electrical effect contributing to the e-DRM was initially investigated on the basis of the intermediates of DRM. On the common DRM catalysts containing La_2_O_3_ and Ni, two intermediates, La_2_O_2_CO_3_ and NiO*_x_* species, are often found, in which the former is due to the CO_2_ adsorption on La_2_O_3_ (CO_2_ + La_2_O_3_ → La_2_O_2_CO_3_) (*54*), while the latter is attributed to the Ni oxidation by CO_2_ (*55*). Therefore, the Ni-La_2_O_3_/AC catalysts after both the e-DRM and the t-DRM at 700°C for 10 hours were characterized by x-ray diffraction (XRD) (Fig. 3A). The fresh catalysts present three phases of metallic Ni, La_2_O_3,_ and La(OH)_3_. There, the La(OH)_3_ species originates from the hydrolysis of La_2_O_3_ with the H_2_O generated in the reduction treatment by H_2_ during the catalyst preparation process (*56*). After the e-DRM, the three phases are maintained, and a minor La_2_O_2_CO_3_ species arises in both the hexagonal and monoclinic structures. However, after the t-DRM, the XRD pattern of the catalyst undergoes substantial changes. First, the two types of La_2_O_2_CO_3_ species completely replace the La_2_O_3_ and La(OH)_3_ species, whose diffraction peaks are much stronger than those after the e-DRM. Second, a diffraction peak at 36.2° is attributed to NiO (1 1 1) (*57*), indicating the formation of NiO*_x_*. Last, some La_3_Ni_2_O_7_ (at 31.8°) and LaNiO_2_ (at 34.4°) species are observed, implying that a portion of NiO*_x_* embeds into the La_2_O_3_ matrix forming composites (*56*).

The oxidation of Ni particles was further investigated by the post–x-ray photoelectron spectroscopy (XPS) (Fig. 3B). Because Ni 2p spectra overlap with La 3d spectra, Ni 3p spectra are used for the investigation of the valence state of Ni. In the fresh catalyst, the Ni^0^ state (66.8 eV) predominates the surface coexisting with 18.9% of the Ni^2+^ state (71.0 eV) (*58*). The proportion of the oxidation state decreases to 10.2% after the e-DRM but increases to 29.7% after the t-DRM, in agreement with the finding based on XRD results (Fig. 3A) that the Ni particles suffer a deeper oxidation by CO_2_ during the t-DRM.

The oxidation level of Ni particles after DRM can be straightforwardly observed by high-angle annular dark field–scanning transmission electron microscopy (HAADF-STEM). Most Ni particles in the Ni-La_2_O_3_/AC catalyst are dispersed on the La_2_O_3_/La(OH)_3_ substrates with uneven sizes ranging from several to more than 100 nanometers (Fig. 3, C to E, and fig. S12). The individual large Ni particles were selected to investigate the change in crystal structure after reactions, due to their ease of observation. The HAADF-STEM image of an individual Ni particle after the e-DRM (Fig. 3C) and the accompanying energy-dispersive x-ray (EDX) spectroscopy analysis (Fig. 3D) show that some La-containing particles (numbered 3 to 6) are in intimate contact with the big Ni particles. Lattice analysis on the area numbered 3 in Fig. 3C and fig. S13 suggests that the La-containing particles are a mixture of La(OH)_3_ and La_2_O_3_, in agreement with the XRD results (Fig. 3A). The lattice analysis on the areas numbered 1 and 2 in Fig. 3C reveals that the edges of the Ni particle retain the continuity of lattice fringe extending from the bulk metal. Regarding an individual Ni particle after the t-DRM (Fig. 3E), its edge contrast is evidently decreased compared to the bulk metal, suggesting that the Ni particle is encapsulated by an oxide layer derived from a clear alignment with the lattice planes of NiO, which is consistent with an EDX observation that the Ni element is surrounded by O element (Fig. 3F). Correspondingly, the lattice analysis on the areas numbered 1 and 2 in Fig. 3E reveals that the oxide layer consists of a few atomic layers of NiO. This finding agrees with the XRD and XPS characterizations after the t-DRM, showing the presence of NiO (Fig. 3A) and the absence of surface Ni^0^ state (Fig. 3B), respectively. These results demonstrate the deep oxidation of Ni particles during the t-DRM.

In addition to NiO formed after the t-DRM, a La_2_O_2_CO_3_ particle is found adhering to the Ni particle by the lattice analysis on the area numbered 3 in Fig. 3E. Great quantities of La_2_O_2_CO_3_ species with a morphology of large pieces of sheets are observed after the t-DRM (Fig. 3G). The HAADF-STEM (Fig. 3, H and I) reveals the distinct, intense spots, indicative of the La atoms within a hexagonal lattice, with weaker spots at centers that represent carbonates, as further evidenced by a corresponding HAADF intensity line scanning in Fig. 3I. This arrangement aligns well with the layered dioxycarbonate structure of hexagonal La_2_O_2_CO_3_ (Fig. 3J). Comparatively, a small quantity of La_2_O_2_CO_3_ sheets are observed after the e-DRM (fig. S14), as their observation is not so prosperous as that observed after the t-DRM, which is consistent with the XRD results (Fig. 3A).

**Fig. 4.**
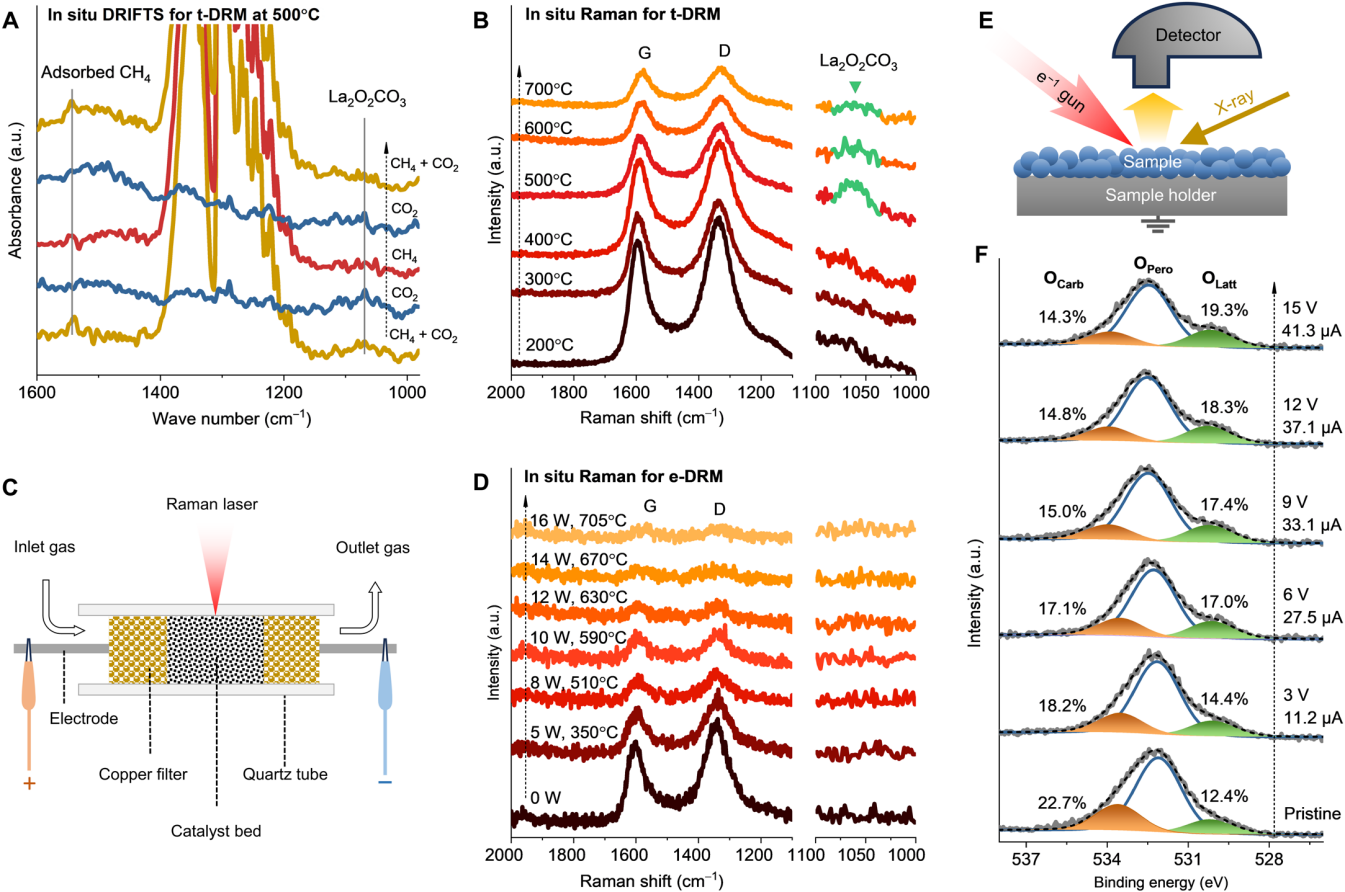
In situ characterizations. (**A**) In situ DRIFTS spectra for the t-DRM over Ni-La_2_O_3_/AC at 500°C during gas switching. (**B**) In situ Raman spectra of t-DRM over Ni-La_2_O_3_/AC with elevated temperature. (**C**) Schematics of in situ Raman characterizations for e-DRM using the homemade reactor. (**D**) In situ Raman spectra of e-DRM over Ni-La_2_O_3_/AC with elevated electric power. (**E**) Schematics of in situ XPS techniques with electrons injected from electron guns. (**F**) In situ O 1s XPS spectra upon applying incremental current with voltages to the Ni-La_2_O_3_/AC catalyst spent after t-DRM. The test samples for in situ DRIFTS and Raman experiments are the fresh Ni-La_2_O_3_/AC catalysts, with a Ni content of 4 wt % and a La/Ni atomic ratio of 2. The sample for the in situ XPS experiment is a Ni-La_2_O_3_/AC catalyst spent after the t-DRM at 700°C for 10 hours, with a Ni content of 10 wt % and a La/Ni atomic ratio of 2.

To confirm the La_2_O_2_CO_3_ species as DRM intermediates, we used in situ diffuse reflectance infrared Fourier transform spectroscopy (DRIFTS) to investigate the t-DRM at 500°C. As shown in Fig. 4A (the full spectra are shown in fig. S15), the infrared bands of adsorbed CH_4_ (1544 cm^−1^) (*59*) and La_2_O_2_CO_3_ (1067 cm^−1^) (*60*) species appear when CH_4_ and CO_2_ are cofed. When the gas is switched to a single CO_2_, the adsorbed CH_4_ disappears; comparatively, when the gas is switched to a single CH_4_, the La_2_O_2_CO_3_ species vanishes. The results confirm the involvement of the La_2_O_2_CO_3_ species in the DRM reactions. The La_2_O_2_CO_3_ intermediate is also detected by in situ Raman characterization for the t-DRM (Fig. 4B). Its characteristic Raman peak at 1064 cm^−1^ (*60*, *61*) appears at 500°C, rightly corresponding to the beginning of the reaction (Fig. 1C), and then the peak becomes weaker at higher temperatures. The in situ Raman characterization for the e-DRM was also performed directly using the homemade reactor, which allows the excitation Raman laser beam to focus on the catalyst through the quartz reactor wall (Fig. 4C). However, the La_2_O_2_CO_3_ species is indistinguishable from the e-DRM, as the electrical power input increases from 0 to 16 W (Fig. 4D), confirming the minor amount of the La_2_O_2_CO_3_ intermediate after the e-DRM. The graphite (G) and defect (D) bands of the AC support decrease in intensity with increasing temperature, which can be attributed to the Raman laser–induced depletion of AC (*62*).

Compared to the t-DRM, the weaker observation of La_2_O_2_CO_3_ in the e-DRM may be attributed to the acceleration of their decomposition due to the electrification. To prove this hypothesis, in situ XPS experiments were conducted using electrons emitted by an electron gun to mimic the current flowing through samples (Fig. 4E) at room temperature (*29*). A Ni-La_2_O_3_/AC catalyst after the t-DRM reaction at 700°C for 10 hours, in which the original La_2_O_3_/La(OH)_3_ phases are nearly completely replaced by La_2_O_2_CO_3_ (Fig. 3A), served as the test sample. Its O 1s XPS spectra can be deconvoluted into three peaks, which are attributed to lattice oxygen (O_Latt_), peroxide (O_Pero_), and carbonate (O_Carb_) (Fig. 4F) (*63*, *64*). Upon applying incremental currents with voltages, the proportion of O_Carb_ decreases, while that of O_Latt_ increases, suggesting the electrically driven decomposition of La_2_O_2_CO_3_ (La_2_O_2_CO_3_ → La_2_O_3_ + CO + O*), releasing CO and oxygen species (O*).

### Reactivity of DRM intermediates

For the Ni-La_2_O_3_ catalyst, both the reduction of NiO*_x_* intermediates and the decomposition of La_2_O_2_CO_3_ intermediates are crucial steps. Sandoval-Diaz *et al.* (*55*) proposed that the Ni catalyst is rapidly deactivated by NiO*_x_* growth and restored with the prompt NiO*_x_* reduction by CH_4_ or H_2_, while the oxygen species from the La_2_O_2_CO_3_ decomposition can oxidize the deposited carbon from CH_4_ dissociation on the active Ni surface to produce CO (*65*–*67*). Accordingly, the promoting roles of electrification on the NiO*_x_* reduction by coke and the La_2_O_2_CO_3_ decomposition for subsequent carbon oxidation were separately tested.

**Fig. 5.**
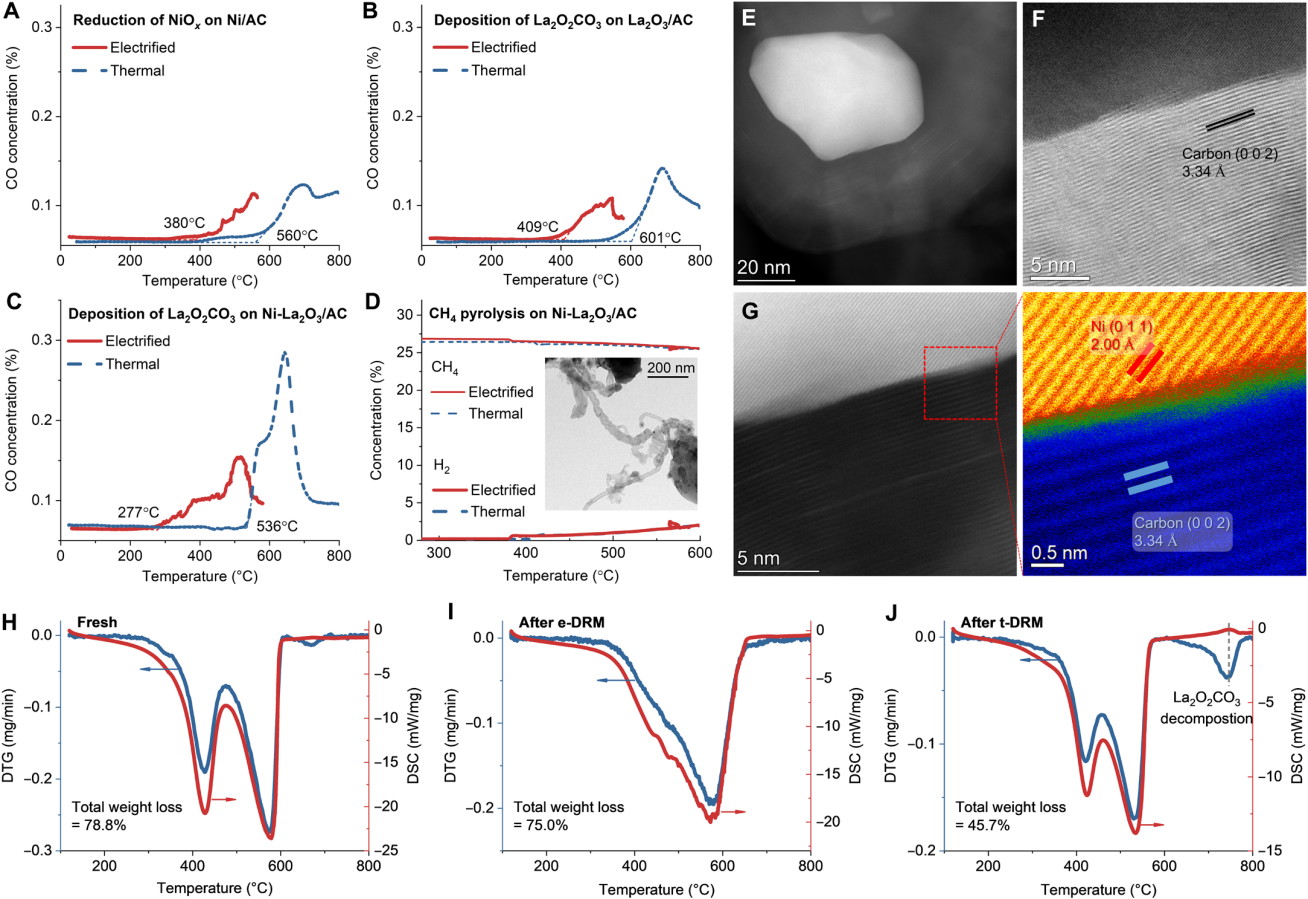
Reactivity of DRM intermediates. (**A**) Reduction of NiO*_x_* over a Ni/AC catalyst with CO generation in electrified EPPR and thermal TPR. The NiO*_x_* species was prefabricated by treating the Ni/AC catalyst under an O_2_ flow at 300°C, with a Ni content of 4 wt %. (**B**) Decomposition of La_2_O_2_CO_3_ over a La_2_O_3_/AC catalyst with CO generation in electrified EPPR and thermal TPR, with a La_2_O_3_ content of 22 wt %. (**C**) Decomposition of La_2_O_2_CO_3_ over the Ni-La_2_O_3_/AC catalyst with CO generation in electrified EPPR and thermal TPR. The La_2_O_2_CO_3_ species was pregenerated by treating the La_2_O_3_/AC and the Ni-La_2_O_3_/AC catalysts under a CO_2_/N_2_ flow at 500°C. (**D**) CH_4_ pyrolysis over the Ni-La_2_O_3_/AC catalyst with H_2_ generation in electrified EPPR and thermal TPR. The inset shows the TEM image of the catalyst after the electrified CH_4_ pyrolysis. (**E** to **G**) HAADF-STEM (E), high-resolution TEM (HRTEM) (F), and magnified HAADF-STEM (G) images of the Ni-La_2_O_3_/AC catalyst spent after the e-DRM. (**H** to **J**) Derivative thermogravimetry (DTG) and differential scanning calorimetry (DSC) curves in air atmosphere for the fresh Ni-La_2_O_3_/AC catalyst (H) and the spent catalysts after the e-DRM (I) and t-DRM (J). The e-DRM was conducted at 16 W for 10 hours, reaching around 700°C, whereas the t-DRM lasted for 10 hours at 700°C. The Ni-La_2_O_3_/AC catalyst has a Ni content of 4 wt % and a La/Ni atomic ratio of 2.

For the NiO*_x_* reduction tests, NiO*_x_* species was prefabricated by oxidizing the Ni/AC catalyst by O_2_ at 300°C as characterized by the XRD characterization (fig. S16). After cooling down to room temperature, the reduction tests of NiO*_x_* were conducted in an Ar flow with the AC support as a reductant (mimicking coke), in an electrified mode and a thermal mode. In the case of thermal tests, a general process of temperature-programmed reaction (TPR) was conducted at a heating rate of 10°C min^−1^; while for electrified tests, a process so-called electrically powered programmed reaction (EPPR) was used, in which the electric power input to the catalyst was linearly increased from 0 to 20 W at a rate of 0.2 W min^−1^, resulting in monotonously increased temperatures (fig. S17). As shown in Fig. 5A and fig. S18, NiO*_x_* reduction is definitely promoted by electrification, as observed by the generation of the CO product becomes evident at about 380° and 560°C in the electrified EPPR and the thermal TPR, respectively.

For the La_2_O_2_CO_3_ decomposition tests, the La_2_O_2_CO_3_ species was preformed on the La_2_O_3_/AC catalyst by treating the catalyst in a CO_2_-containing flow at 500°C as characterized by the post-XRD characterization (fig. S19). After cooling down to room temperature under a N_2_ flow, the decomposition of La_2_O_2_CO_3_ was conducted in both the TPR and EPPR processes. As shown in Fig. 5B, the CO product, which is generated directly from the La_2_O_2_CO_3_ decomposition and the following oxidation of AC by the released oxygen species, starts to become obvious at ~409°C in the EPPR, 192°C lower than that in the thermal TPR (601°C). This suggests that the electrification can markedly accelerate the decomposition of the La_2_O_2_CO_3_ intermediate and promote coke oxidation, agreeing with the in situ XPS results (Fig. 4F).

The synergy between La_2_O_3_ and Ni particles for the La_2_O_2_CO_3_ decomposition was further studied. As shown in Fig. 5C, either thermal or electrical decomposition of La_2_O_2_CO_3_ on the Ni-La_2_O_3_/AC catalyst shifts to lower temperatures and forms more CO product compared with that on the La_2_O_3_/AC (Fig. 5B), suggesting the promoting role of Ni for the decomposition of La_2_O_2_CO_3_. Furthermore, the electrical effect is more pronounced in the presence of Ni, as the temperatures of the La_2_O_2_CO_3_ decomposition are 277° and 536°C in EPPR and TPR, respectively, and the difference between them is 259°C (Fig. 5C), while that is 192°C in the absence of Ni (Fig. 5B). Mutually, the presence of La_2_O_3_ promotes the oxidation of coke on Ni particles derived from CH_4_ pyrolysis because the decomposition of La_2_O_2_CO_3_ species originated from CO_2_ adsorption on La_2_O_3_ offers oxygen species to consume the deposited carbon on Ni particles (*68*). It can be expected that, if the CO_2_ feeding or the adjacent La_2_O_3_ is absent, then the deposited carbon will accumulate and form coke, deactivating the catalyst. In the first case, namely, without CO_2_ feeding, the CH_4_ pyrolysis into H_2_ over the Ni-La_2_O_3_/AC catalyst is weak (Fig. 5D), with the formation of carbon nanotubes (CNTs) in tip growth mode (*69*) as observed by the postcharacterizations of TEM (Fig. 5D inset) and scanning electron microscopy (SEM) (fig. S20A). In the second case, namely, without adjacent La_2_O_3_, the coke formation on the surface of the Ni metal particles is evident. As observed in the HAADF-STEM and high-resolution TEM (HRTEM) images in Fig. 5 (F and G), after the e-DRM over the Ni-La_2_O_3_/AC catalyst, an isolated Ni metal particle is detected to be surrounded by deposited graphitic layers.

Hence, the coke on the Ni-La_2_O_3_/AC catalyst after DRM was checked. Unlike the CH_4_ pyrolysis, the post-SEM characterizations reveal no CNT formation after the e-DRM (fig. S20B) and the t-DRM (fig. S20C), suggesting the markedly suppressed coking. The coking was further assessed by thermogravimetry/derivative thermogravimetry/differential scanning calorimetry (TG/DTG/DSC) in airflow (*70*–*72*). The TG curves are shown in fig. S21, while the DTG and DSC curves are presented in Fig. 5 (H to J). For the fresh sample, the total weight loss is 78.8%, and the major weight loss occurs at below 700°C synchronously with DSC exothermic peaks (Fig. 5H), due to the oxidation of the AC support during the TG process. The catalyst after the e-DRM also undergoes an exothermic weight loss process, yet the weight loss is integrally shifted toward higher temperatures (fig. S21), which may originate from the enhanced graphitization of the AC support by the electrification process. However, the total weight loss (75.0%) remains comparable to that of the fresh catalyst, confirming negligible coke formation and AC gasification during the e-DRM. Regarding the catalyst after the t-DRM, in addition to exothermic weight losses occurring below 600°C, a distinct weight loss accompanied by a DSC endothermic peak is identified at about 750°C (Fig. 5J), which is derived from the decomposition of the accumulated La_2_O_2_CO_3_ species during the t-DRM (Figs. 3A and 4, A and B). Furthermore, the total weight loss (45.7%) is markedly lower than that of the fresh catalyst, which can be attributed to the AC gasification by CO_2_ on the deactivated t-DRM catalyst (Fig. 1D).

### Mechanism for e-DRM

**Fig. 6.**
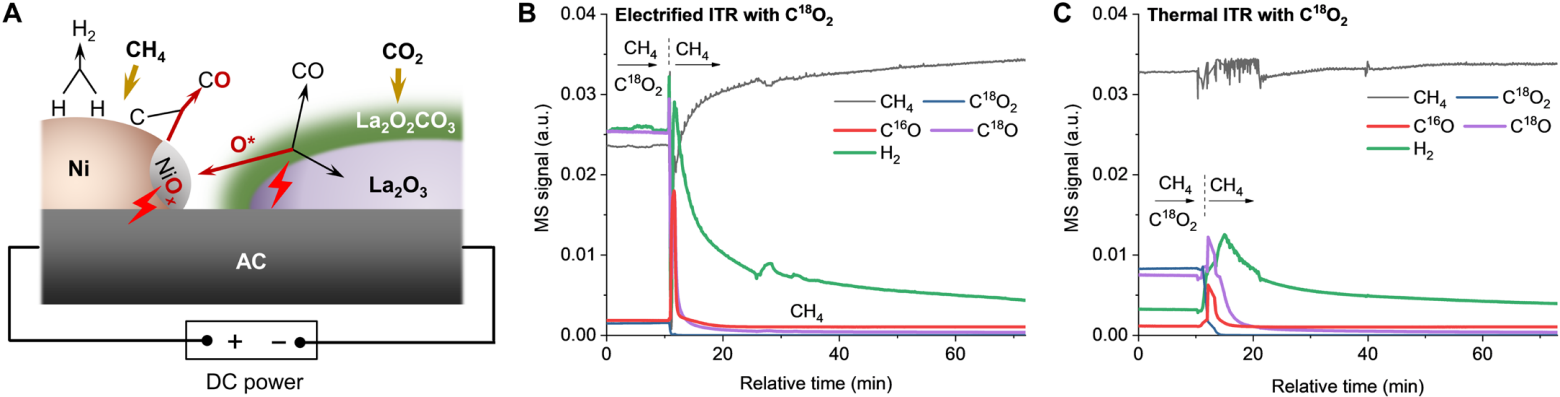
e-DRM mechanism. (**A**) Mechanistic diagram for e-DRM. (**B**) Evolutions of the electrified ITP over Ni-La_2_O_3_/AC at 16 W. MS, mass spectrometry. (**C**) Evolutions of the thermal ITP over Ni-La_2_O_3_/AC at 700°C. The Ni-La_2_O_3_/AC catalyst has a Ni content of 4 wt % and a La/Ni atomic ratio of 2. ITP, isotopic transient response.

Until now, the e-DRM mechanism can be schematically illustrated in Fig. 6. Initially, CO_2_ adsorbs on La_2_O_3_ forming La_2_O_2_CO_3_ species, which decompose into CO product and oxygen species under electrical stimulation. Meanwhile, CH_4_ dissociates on the active Ni surface, generating H_2_ product with carbon being deposited. Subsequently, the released oxygen is transferred to the active Ni surface, forming the NiO*_x_* species. Last, the NiO*_x_* is electrically driven to react with the deposited carbon on the active Ni surface, forming CO product. The synergy between Ni and La_2_O_3_ prevents carbon deposition from bonding together into coke and inhibits metallic Ni deep oxidation (*67*). Throughout the entire process, the electrification of the DRM process plays a pivotal role in accelerating oxygen circulation, which is similar to the electrical effect derived from the electrically driven release of lattice oxygen, as found in our previous studies on electrified soot combustion (*24*–*26*). In the e-DRM, the electrical effect activates the lattice oxygen of La_2_O_2_CO_3_ and NiO*_x_*, promoting the La_2_O_2_CO_3_ decomposition, releasing oxygen species, and the further oxidation of deposited carbon via the reduction of NiO*_x_*, inhibiting the coking from CH_4_ pyrolysis. In sharp contrast, the corresponding procedure is rather sluggish for the t-DRM counterpart, leading to the accumulation of the La_2_O_2_CO_3_ species and the deep oxidation of Ni particles and their combination with nearby La_2_O_3_, which deactivates La_2_O_3_ and Ni for activation of CO_2_ and CH_4_, respectively.

The proposed mechanism was corroborated by designed electrified and thermal isotopic transient response (ITR) experiments, in which the steady-state e-DRM and t-DRM reactions with CH_4_ + C^18^O_2_ feed were perturbed by discontinuing C^18^O_2_ feed. In the electrified ITR (Fig. 6B), the cessation of C^18^O_2_ causes a swift drop of C^18^O evolution, while the H_2_ evolution declines slowly, implying that CH_4_ pyrolysis is occurring continually. In particular, there is a C^16^O spike during transient switching, which can be attributed to the oxidation of the CH*_x_* and/or coke by the catalyst’s native lattice oxygen. In the thermal ITR (Fig. 6C), conversely, the H_2_ evolution surges when the C^18^O_2_ feed is halted, which is derived from the reduction of the NiO*_x_* intermediate to metallic Ni by the CH*_x_* and/or coke, thus restoring the activity for CH_4_ dissociation. Moreover, a wide peak of C^18^O emerges alongside a C^16^O peak during transient switching, corroborating the accumulation of a greater quantity of ^18^O_2_-containing intermediates (NiO*_x_* and La_2_O_2_CO_3_). The involvement of the lattice oxygen suggests that both the e-DRM and t-DRM follow a Mars-van Krevelen (MvK)–type mechanism, at least partially (*32*). Assuming that both the MvK and Langmuir-Hinshelwood (L-H) mechanisms coexist in the DRM reactions, we derived a formula to calculate the contribution of the MvK pathway (μ_MvK_) in the two DRM reactions based on the ITR results (note S2). The calculated μ_MvK_ for the e-DRM (16.1%) is lower than that for t-DRM (29.5%). This is consistent with the accelerating role of the electrically driven release of lattice oxygen in the intermediate conversion of the e-DRM, which alleviates their accumulation into the bulk structure and promotes their participation in the DRM reactions via the L-H pathway.

To investigate the universality of the electricity-induced improvement in DRM, two additional catalysts were prepared. Specifically, the La_2_O_3_ promoter in the Ni-La_2_O_3_/AC catalyst was substituted with CeO_2_ and MgO, resulting in the Ni-CeO_2_/AC and Ni-MgO/AC catalysts, respectively. Both the e-DRM and t-DRM performances of these catalysts were evaluated. By comparison, the improvement through the electricity of the Ni-CeO_2_/AC is notable, as the e-DRM performance is substantially higher than that of the t-DRM counterpart (fig. S22A). In contrast, the improvement observed over the Ni-MgO/AC is not so evident (fig. S22B). This can be attributed to the fact that the lattice oxygen in CeO_2_ is labile (*73*), while that in MgO is relatively inert. As reported by Nakano *et al.* (*74*), who focus on electrical field–enhanced DRM reactions, the lattice oxygen of CeO_2_ can be activated by an electric field to facilitate the activation and dehydrogenation of CH_4_ in DRM. This finding further evidences the promoting role of electrically driven release of lattice oxygen in DRM reactions.

## DISCUSSION

An electrification technology for DRM has been developed by passing electric current through the conductive Ni-La_2_O_3_/AC catalyst under several to tens of voltages in the homemade electrified reactor. The e-DRM outperforms the conventional t-DRM in CO_2_ and CH_4_ conversions and H_2_/CO ratio especially at above 600°C. The e-DRM performance is nearly at thermodynamic equilibrium, while the t-DRM performance decelerates with the increase in temperature. In contrast with the sharp drop of the t-DRM performance within 10 hours due to severe oxidation of active Ni particles and accumulation of La_2_O_2_CO_3_ intermediates, the e-DRM only experiences a slight decrease during the initial 10 hours and then maintains a CO_2_ conversion of 77% and a H_2_/CO ratio of 0.9 for at least 120 hours. Notably, the energy efficiency reaches 2.976 mmol kJ^−1^ at the gram level of catalysts, outclassing that of t-DRM and surpassing previously reported values using plasma and pulsed laser strategies. The high energy efficiency leads to a substantial decrease in CO_2_ emissions, enabling the achievement of net CO_2_-negative emission when powered by common low-carbon and sustainable electricity. Mechanistic studies reveal that the electrically driven release of lattice oxygen plays vital roles in the e-DRM, which not only enhances the decomposition of the La_2_O_2_CO_3_ species generated from CO_2_ adsorption on La_2_O_3_ but also promotes the reduction of the resultant NiO*_x_* species for the oxidation of deposited carbon, namely, bridging the synergy between Ni and La_2_O_3_. Moreover, the preserved surface metallic state of Ni blocks the combination of CH_4_-dissociated H atoms (H*) with surface oxygen to form hydroxyl (OH^−^) and H_2_O, thereby effectively inhibiting RWGS. This electrification technology offers a clean solution to overcome the stumbling block of DRM, namely, net CO_2_-positive emission, motivating DRM from laboratory to industrialization. In the future, we will fabricate conductive catalysts on which Ni metal particles are highly dispersed on La_2_O_3_ to ensure an efficacious synergy between them while exploring oxidation-resistant supports as AC alternatives to prevent catalyst degradation from CO_2_ overoxidation.

## MATERIALS AND METHODS

### Catalyst preparation

The Ni-La_2_O_3_/AC catalysts were prepared using an incipient wetness impregnation method. The salts of Ni(NO_3_)_2_·6H_2_O (AR grade, Sinopharm) and La(NO_3_)_3_·6H_2_O (AR grade, Aladdin) were dissolved in deionized water. A coconut shell–based granular activated carbon (12 to 30 mesh, Jacobi) with a weight of 5.0 g was impregnated in the solution to yield catalysts with 1 to 5 wt % of Ni content (where weight % represents the proportion of Ni relative to the total solid) and a La/Ni atomic ratio of 0 to 3. Afterward, the mixture was treated with ultrasound for 1 hour, sealed, and aged at room temperature overnight. Then, the sample was dried at 80°C overnight and calcined at 850°C for 5 hours in a pure N_2_ flow (20 ml min^−1^). After that, the sample was reduced in a pure H_2_ flow (20 ml min^−1^) at 600°C for 2 hours, obtaining the final Ni-La_2_O_3_/AC catalysts.

The La_2_O_3_ promoter in the Ni-La_2_O_3_/AC catalyst was substituted with CeO_2_ and MgO, yielding two additional catalysts: Ni-CeO_2_/AC and Ni-MgO/AC, respectively. Their preparation procedures are analogous to that of Ni-La_2_O_3_/AC, except that La(NO_3_)_3_·6H_2_O was replaced by Ce(NO_3_)_3_·6H_2_O (AR grade, McLean) and Mg(NO_3_)_2_·6H_2_O (AR grade, Sinopharm) as the precursors for CeO_2_ and MgO, respectively. The Ni contents of the two catalysts are 4 wt %, while both the atomic ratios of Ce/Ni and Mg/Ni are 2.

A control catalyst that Ni/La_2_O_3_ without AC supports was prepared by wetness impregnation methods. A La_2_O_3_ powder was obtained via calcining La(NO_3_)_3_·6H_2_O salts (AR grade, Aladdin) at 800°C for 2 hours. Then, the powder was impregnated into an aqueous solution of Ni(NO_3_)_2_ (AR grade, Aladdin). The mixture was stirred at 80°C for 3 hours, followed by overnight drying at the same temperature and calcining at 600°C for 2 hours. Afterward, the sample was sieved into a particle size range of 40 to 60 mesh and subsequently reduced in a pure H_2_ flow (20 ml min^−1^) at 600°C for 2 hours, obtaining the Ni/La_2_O_3_ catalyst with 4 wt % Ni content.

### Catalyst characterizations

The crystalline phases of catalysts were identified by powder XRD with a Bruker D8 Advance Davinci diffractometer using Cu-Kα radiation (λ =1.5418 Å) and operating at 40 kV and 40 mA. The surface chemical states of catalysts were analyzed by XPS measurements (Kratos Axis Ultra DLD) with an Al Kα radiation source at room temperature and under a vacuum of 10^−7^ Pa. Binding energies were calibrated using C 1s at 284.4 eV as the standard. Probe spherical aberration–corrected HAADF-STEM and HRTEM with EDX mapping were carried out on a Thermo Fisher Spectra 300 instrument. The micromorphology was also observed by SEM characterizations (Hitachi S-4800). TG/DTG/DSC analysis of a sample of about 5 mg was conducted in a NETZSCH STA449F3 measurement under an airflow (50 ml min^−1^) at a heating rate of 10°C min^−1^ from room temperature to 800°C.

### Assembly of reactor for e-DRM

A homemade reactor for e-DRM was constructed as shown in Fig. 1A. The Ni-La_2_O_3_/AC catalyst pellets are sandwiched between two current collectors, which were composed of filters prepared from sintered copper powder, and the assembly was placed in the middle of a quartz tube with an inner diameter of 6 mm. Two separate stainless steel electrodes were fixed in contact with the two filters and connected to an adjustable DC power (KORAD KA6003P, Shenzhen, China), forming a catalyst-containing circuit to supply electric power to the catalyst. The reactor is wrapped with a cotton jacket to preserve heat. To get the accurate temperature of the catalyst layer, six temperature points of the reactor were measured by calibrated K-type thermocouples (Fig. 1A). Among them, thermocouples 1 to 5 are arranged on the outside surface of the quartz tube in the order from inlet to outlet: Thermocouples 1 and 5 correspond to the outside faces of the inlet and outlet copper filters, respectively; thermocouples 2 and 4 correspond to the inlet and outlet faces of the catalyst layer, respectively; thermocouple 3 corresponds to the center of the catalyst layer. Thermocouple 6 is inserted into the electrode that is positioned near the outlet, which is a tube with a closed end in contact with the copper filter. The tip of the thermocouple extends to the closed end of the tube electrode.

### Performance evaluation for e-DRM

For a typical e-DRM, 0.2 g of the Ni-La_2_O_3_/AC catalyst was packed into the homemade reactor (Fig. 1A). Before the reaction, the catalyst was purged for 30 min by pure He flow at a rate of 30 ml min^−1^, which is controlled by mass flowmeters (D077, Qixing Huachuang), to remove the air in the reactor. A mixture of CH_4_ and CO_2_ with He carrier was introduced to the catalyst GHSVs within 15 to 27 liters hour^−1^ g_cat_^−1^. The CO_2_:CH_4_ feed ratio was varied from 1:1 to 1:3, while the total concentration of the two gasses was kept at 40 vol %. When the concentrations of reaction gasses were stabilized, a series of electric powers from 8 to 16 W was supplied to the Ni-La_2_O_3_/AC catalyst under an upper voltage output of 60 V and an upper current output of 3 A, resulting in the electric current flowing through the catalyst. The generated Joule heating elevated the catalyst temperature and triggered the DRM. The outlet gas was analyzed by an online gas chromatograph (Agilent 8890) equipped with two thermal conductivity detectors. For comparison, the traditional t-DRM was also implemented by directly heating the homemade reactor at 500° to 700°C in a tube oven, using the same catalyst and reaction conditions.

The amplified e-DRM was conducted using 5.4 g of the same catalyst within a similar electrified reactor with an inner diameter of 18 mm. The GHSVs for CH_4_ and CO_2_ flows were the same as those of the unamplified e-DRM, which were 5.6 and 2.8 liters hour^−1^ g_cat_^−1^, respectively. The conversions of CH_4_ (*X*_CH4_) and CO_2_ (*X*_CO2_), H_2_/CO ratio (*R*_H2/CO_), and carbon balance (*B*_carbon_) were calculated as follows

$$X_{CH_{4}}\left(\%\right)=\frac{F_{CH_{4,\text{in}}}-F_{CH_{4,\text{out}}}}{F_{CH_{4,\text{in}}}}\times 100\%$$ (3)

$$X_{CO_{2}}\left(\%\right)=\frac{F_{CO_{2,\text{in}}}-F_{CO_{2,\text{out}}}}{F_{CO_{2,\text{in}}}}\times 100\%$$ (4)

$$R_{H_{2}/\text{CO}}=\frac{F_{H_{2,\text{out}}}}{F_{{\text{co}}_{,\text{out}}}}$$ (5)

$$B_{\text{carbon}}\left(\%\right)=\frac{F_{CO_{,\text{out}}}}{\left(F_{CH_{4,\text{in}}}-F_{CH_{4,\text{out}}}\right)+\left(F_{CO_{2,\text{in}}}-F_{CO_{2,\text{out}}}\right)}\times 100\%$$ (6)

where *F*_CH4,in_ and *F*_CO2,in_ represent the volume flow rate of inlet CH_4_ and CO_2_ in the inlet gas, respectively, while *F*_CH4,out_, *F*_CO2,out_, *F*_H2,out_, and *F*_CO,out_ are the volume flow velocities of CH_4_, CO_2_, H_2_, and CO in the inlet gas, respectively.

### Isotopic e-DRM reaction with ^13^CH_4_ and ^13^CO_2_

The Ni-La_2_O_3_/AC catalyst weighing 200 mg was packed into the homemade reactor, and the catalyst was purged for 30 min by pure Ar flow at a rate of 100 ml min^−1^ to remove the air in the reactor. Then, the gas flow was switched to a mixture comprising 0.67 vol % ^13^CH_4_ and 0.33 vol % ^13^CO_2_, with Ar as the balance gas, at a total flow rate of 150 ml/min. Electric power was input to the catalyst and linearly increased from 0 to 16 W at a rate of 0.2 W min^−1^ and subsequently maintained at 16 W for the e-DRM reaction. The effluent gas was analyzed by a mass spectrometer (OmniStar 200, Pfeiffer Vacuum).

### Isotopic transient response experiment with C^18^O_2_

The Ni-La_2_O_3_/AC catalyst weighing 200 mg was packed into the homemade reactor, and the catalyst was purged for 30 min by pure Ar flow at a rate of 100 ml min^−1^ to remove the air in the reactor. Then, the gas flow was switched to a mixture comprising 2 vol % CH_4_ and 1 vol % C^18^O_2_, with Ar as the balance gas, at a total flow rate of 102 ml/min. The electric power was input to the catalyst and linearly increased from 0 to 16 W at a rate of 0.2 W min^−1^ and subsequently maintained at 16 W for the e-DRM reaction. Once the reaction reached a steady state, the C^18^O_2_ feed was replaced with pure Ar while maintaining the same flow rate. Following ~1 hour of reaction, the C^18^O_2_ feed was restored. The effluent gas was analyzed by the mass spectrometer. For comparison, the same dynamic gas switching protocol was also implemented for the t-DRM counterpart at 700°C.

### Tests for the decomposition of La_2_O_2_CO_3_

The Ni-La_2_O_3_/AC or La_2_O_3_/AC catalyst weighing 50 mg was packed into the homemade reactor and pretreated at 300°C in N_2_ flow (60 ml min^−1^) for 30 min. For the generation of La_2_O_2_CO_3_, the gas flow was switched to 13.3 vol % CO_2_ balanced by N_2_ (60 ml min^−1^) and heated at 500°C for 1.5 hours. At the same temperature, the catalyst was purged by Ar (70 ml min^−1^) to remove the weakly adsorbed CO_2_ and then cooled down to room temperature, ready for the decomposition test of La_2_O_2_CO_3_. In the electrified EPPR test, the electric power was supplied to the sample and linearly increased from 0 to 20 W at a rate of 0.2 W min^−1^ in the Ar flow. Regarding the thermal TPR test, the sample was heated from room temperature to 800°C at a rate of 10°C min^−1^ in the Ar flow. The effluent gas was analyzed by a mass spectrometer (OmniStar 200, Pfeiffer Vacuum).

### Tests for the reduction of NiO_x_

The unreduced Ni/AC catalyst weighing 50 mg was packed into the homemade reactor, exposed to an O_2_ flow (20 ml min^−1^), and heated at 300°C for 2 hours, obtaining NiO*_x_* species on the Ni/AC catalyst. When the temperature was cooled down to room temperature, the sample was purged by Ar (70 ml min^−1^), ready for the reduction test of NiO*_x_* by the AC support. In the electrified EPPR test, the electric power was supplied to the sample and linearly increased from 0 to 20 W at a rate of 0.2 W min^−1^ in the Ar flow. Regarding the thermal TPR test, the sample was heated from room temperature to 800°C at a rate of 10°C min^−1^ in the Ar flow. The effluent gas was analyzed by the mass spectrometer.

### Tests for pyrolysis of CH_4_

The pyrolysis of CH_4_ on Ni-La_2_O_3_/AC catalysts was studied. Initially, 0.2 g of Ni-La_2_O_3_/AC catalyst was packed into the homemade reactor and purged by an Ar flow (70 ml min^−1^) for 30 min to remove the air in the reactor, ready for testing. The gas flow was switched to 26.7 vol % CH_4_ balanced by Ar (51 ml min^−1^). The pyrolysis tests were carried out in electrified and thermal modes. For the electrified tests, a process of EPPR was used, in which the electric power input to the catalyst was linearly increased from 0 to 20 W at a rate of 0.1 W min^−1^. Regarding the thermal tests, a general process of TPR was carried out, in which the catalyst was heated to 800°C from room temperature at a rate of 4.5°C min^−1^. The effluent gas was analyzed by the mass spectrometer.

### In situ DRIFTS experiments

In situ DRIFTS measurements for t-DRM were conducted on a Fourier transform infrared spectrometer (VERTEX 70v, Bruker). Before the t-DRM, the Ni-La_2_O_3_/AC catalyst was placed in the DRIFTS cell and purged in a N_2_ flow (40 ml min^−1^) at 500°C for 30 min to remove adsorbed water and most of the CO_2_ from air exposure. Then, after recording the background spectrum in N_2_ flow at 500°C, the gas flow was switched to a DRM reaction gas containing 9 vol % CH_4_ and 4.5 vol % CO_2_ mixture balanced by N_2_ (44 ml min^−1^) until the DRIFTS spectra were stabilized. Subsequently, the CH_4_ and CO_2_ feeds were alternatively halted, and lastly, the DRM reaction gas was resumed. During the process, the DRIFTS spectra were recorded every 60 s from 4000 to 800 cm^−1^ with 64 accumulated scans and a resolution of 4 cm^−1^.

### In situ Raman experiments

The Ni-La_2_O_3_/AC catalyst during the DRM was in situ characterized by Raman spectroscopy (Renishaw inVia Reflex). For the e-DRM, the homemade reactor can be easily repurposed as an in situ cell setup on the Raman spectrometer (Fig. 4C). The excitation laser (532 nm) with a power of 1.2 mW can penetrate through the wall of the quartz tube to focus on the surface of the catalyst pellets, enabling the acquisition of Raman spectra. Hence, the e-DRM was carried out as described above, with 50 mg of the Ni-La_2_O_3_/AC catalyst and 21 liters hour^−1^ g_cat_^−1^ of GHSV, during which the Raman spectra were collected in situ under different electric power inputs from 0 to 16 W. For the t-DRM, a commercial in situ cell for Raman characterization was used, in which 50 mg of the Ni-La_2_O_3_/AC catalyst was loaded, and the reaction gas was introduced with 21 liters hour^−1^ g_cat_^−1^ of GHSV. The catalyst was heated at 200° to 700°C for DRM, and the Raman spectra were collected in situ.

### In situ XPS experiments

The Ni-La_2_O_3_/AC catalyst after the t-DRM reaction at 700°C for 10 hours was selected as a test sample, with La_2_O_2_CO_3_ predominantly replacing the original La_2_O_3_/La(OH)_3_ phases. The in situ XPS experiment was conducted by the Kratos Axis Ultra DLD XPS system equipped with an electron gun, which can inject electrons into the sample to mimic current (Fig. 4E). Under vacuum conditions, the positive bias voltage of the electron gun from 3 to 15 V was applied at room temperature, and the XPS spectra of the sample were recorded in situ.

## Supplementary Materials

### This PDF file includes:

Figs. S1 to S22 Notes S1 and S2 Tables S1 to S3
