# Supplementary material for: Electrified dry reforming of methane on Ni-La2O3–loaded activated carbon: A net CO2-negative reaction
Source: Sci Adv. 2025 Jul 11;11(28):eadv1585. doi: 10.1126/sciadv.adv1585 (PMC12248285; doi:10.1126/sciadv.adv1585)
Supplement: Supplementary file 1 — Figs. S1 to S22 Notes S1 and S2 Tables S1 to S3 [file sciadv.adv1585_sm.pdf]

Science Advances
AAAS

Supplementary Materials for

Electrified dry reforming of methane on Ni-La_2O_3–loaded activated carbon: A net CO_2-negative reaction

Wei Zhao *et al.*

Corresponding author: Yexin Zhang, zhangyexin@nimte.ac.cn; Zhaoliang Zhang, chm_zhangzl@ujn.edu.cn; Jian Zhang, jzhang@nimte.ac.cn

Sci. Adv. **11**, eadv1585 (2025)
DOI: 10.1126/sciadv.adv1585

This PDF file includes:

Figs. S1 to S22
Notes S1 and S2
Tables S1 to S3

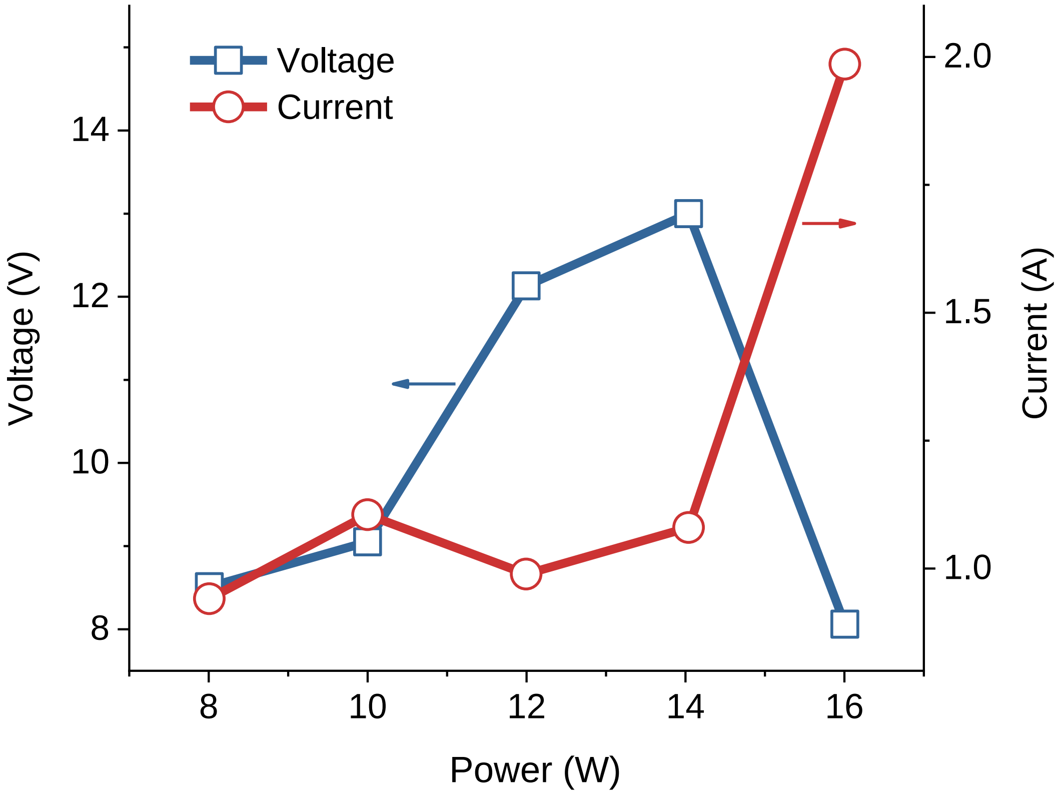

Fig. S1. Plots of voltage and current as a function of electric power for e-DRM. The e-DRM was performed over Ni-La_2O_3/AC with a CH_4/CO_2 ratio of 2 and a GHSV of 21 L h^{-1} g_{cat}^{-1}. The Ni-La_2O_3/AC catalyst has a Ni content of 4 wt.% and a La/Ni atomic ratio of 2.

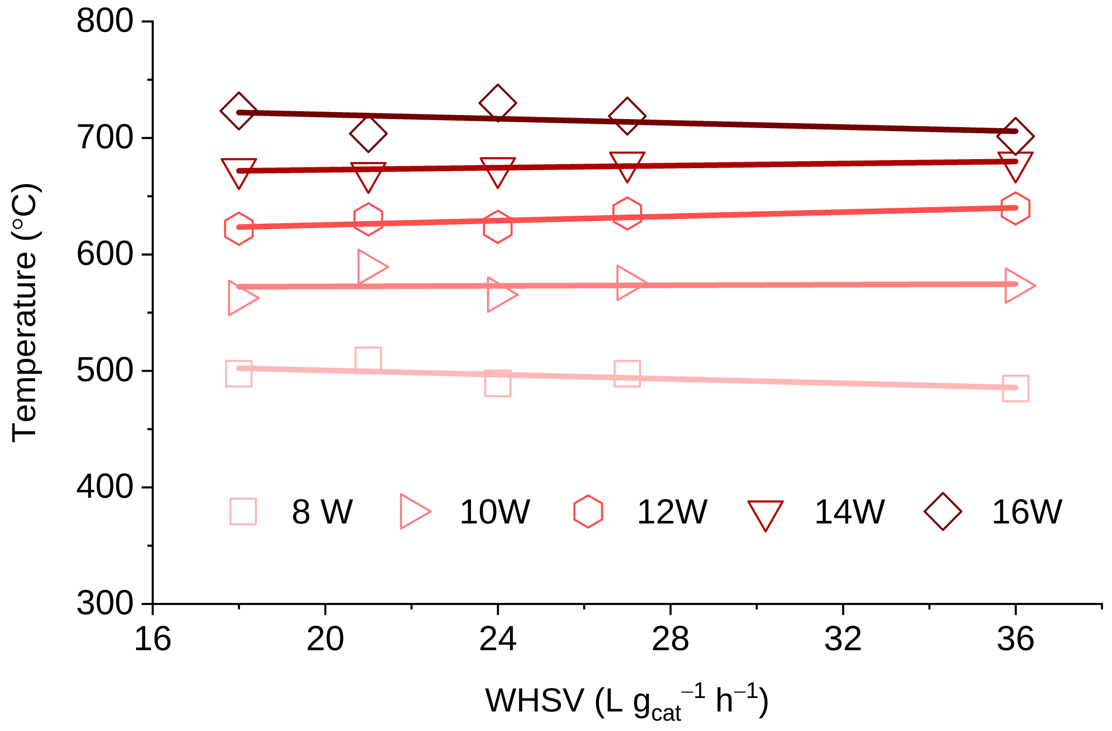

Fig. S2. Temperatures vs. GHSV with different electric powers for the e-DRM over Ni-La_2O_3/AC. The Ni-La_2O_3/AC catalyst has a Ni content of 4 wt.% and a La/Ni atomic ratio of 2.

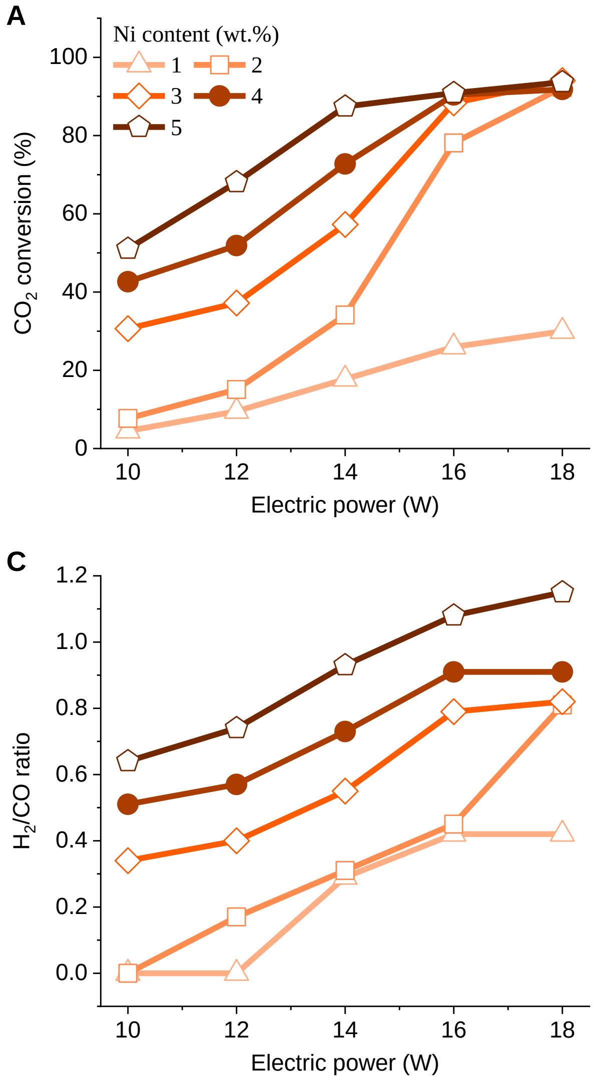

Fig. S3. Optimization of Ni content of Ni-La_2O_3/AC for e-DRM. (**A**) CO_2 conversion as a function of electric power. (**B**) CH_4 conversion as a function of electric power. (**C**) H_2/CO ratio as a function of electric power. (**D**) Carbon balance as a function of electric power. The reactions were conducted with a CH_4/CO_2 ratio of 2 and a GHSV of 21 L h^{-1} g_{cat}^{-1}, and the La/Ni atomic ratio of the Ni-La_2O_3/AC catalyst.

The Ni contents in the Ni-La_2O_3/AC catalysts were optimized within a range of 1–5 wt.%. When it is 1 wt.%, the e-DRM performance is inferior, only with 30% of CO_2 conversion and 0.42 of H_2/CO ratio as the power is elevated to 18 W. The performance is improved by the increase in Ni content. When the Ni contents are between 3–5 wt.%, the CO_2 conversion reaches a similar value (around 90%) when the power is elevated up to 16 W. However, the carbon balance with 5 wt.% of Ni content suddenly drops to 69% at 18 W. By balance, the optimal Ni content of is 4 wt.% for the Ni-La_2O_3/AC catalysts.

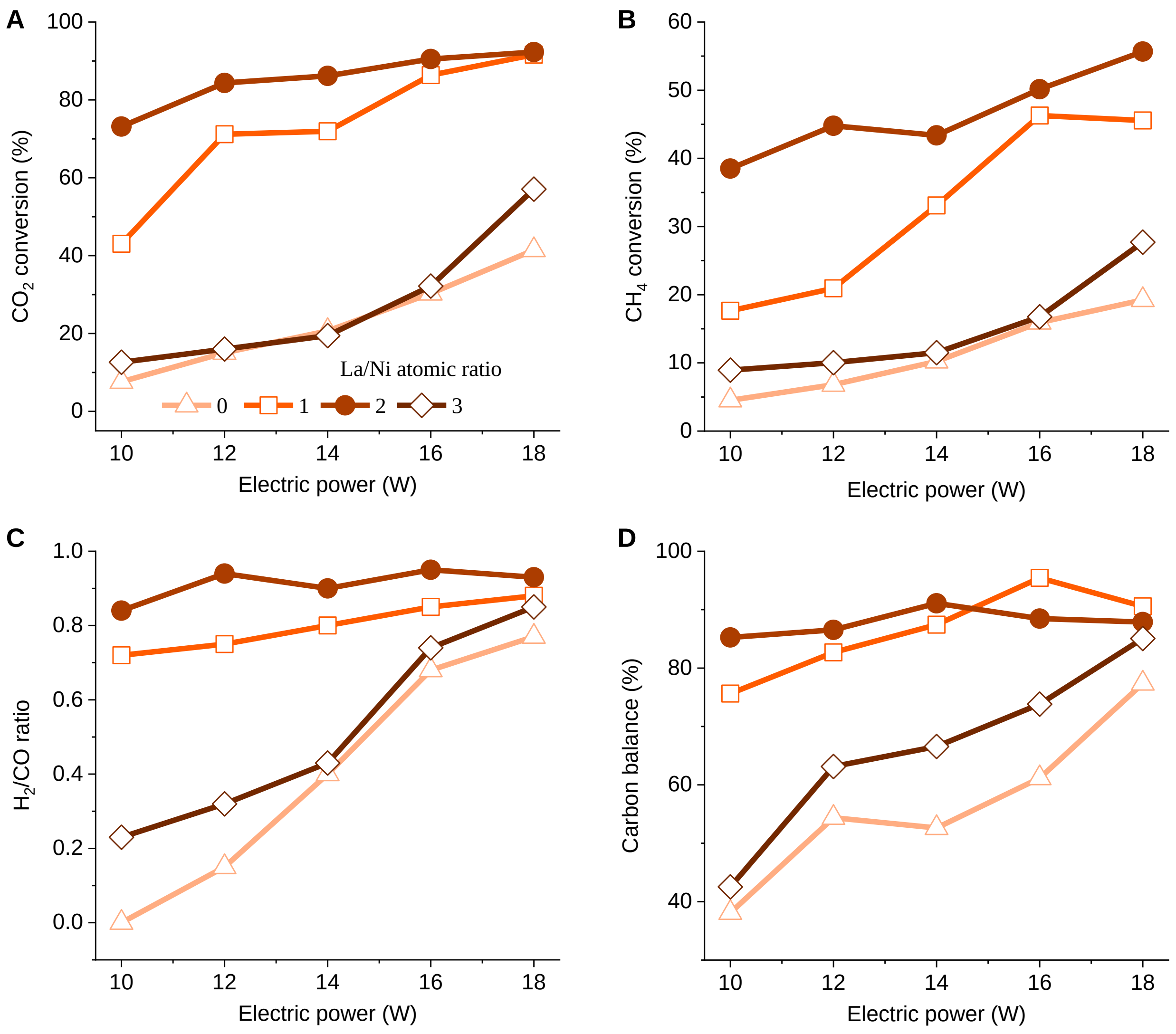

Fig. S4. Optimization of La/Ni atomic ratio of Ni-La_2O_3/AC for e-DRM. (**A**) CO_2 conversion as a function of electric power. (**B**) CH_4 conversion as a function of electric power. (**C**) H_2/CO ratio as a function of electric power. (**D**) Carbon balance as a function of electric power. The reactions were conducted with a CH_4/CO_2 ratio of 2 and a GHSV of 21 L h^{-1} g_{cat}^{-1}, and the Ni content of the Ni-La_2O_3/AC catalyst is 4 wt.%.

The La/Ni atomic ratios of the Ni-La_2O_3/AC catalysts were optimized within a range of 0–3. The CO_2 conversion and the H_2/CO ratio are obviously improved as the ratio increases from 0 to 2, but the both suddenly drop when the ratio is raised to 3. Among the La/Ni atomic ratios, the ratio of 2 delivers the best e-DRM performance with relatively stable carbon balance (around 90%), indicating the less carbon deposition. Therefore, the optimal La/Ni atomic ratio is determined to be 2 for the Ni-La_2O_3/AC catalysts.

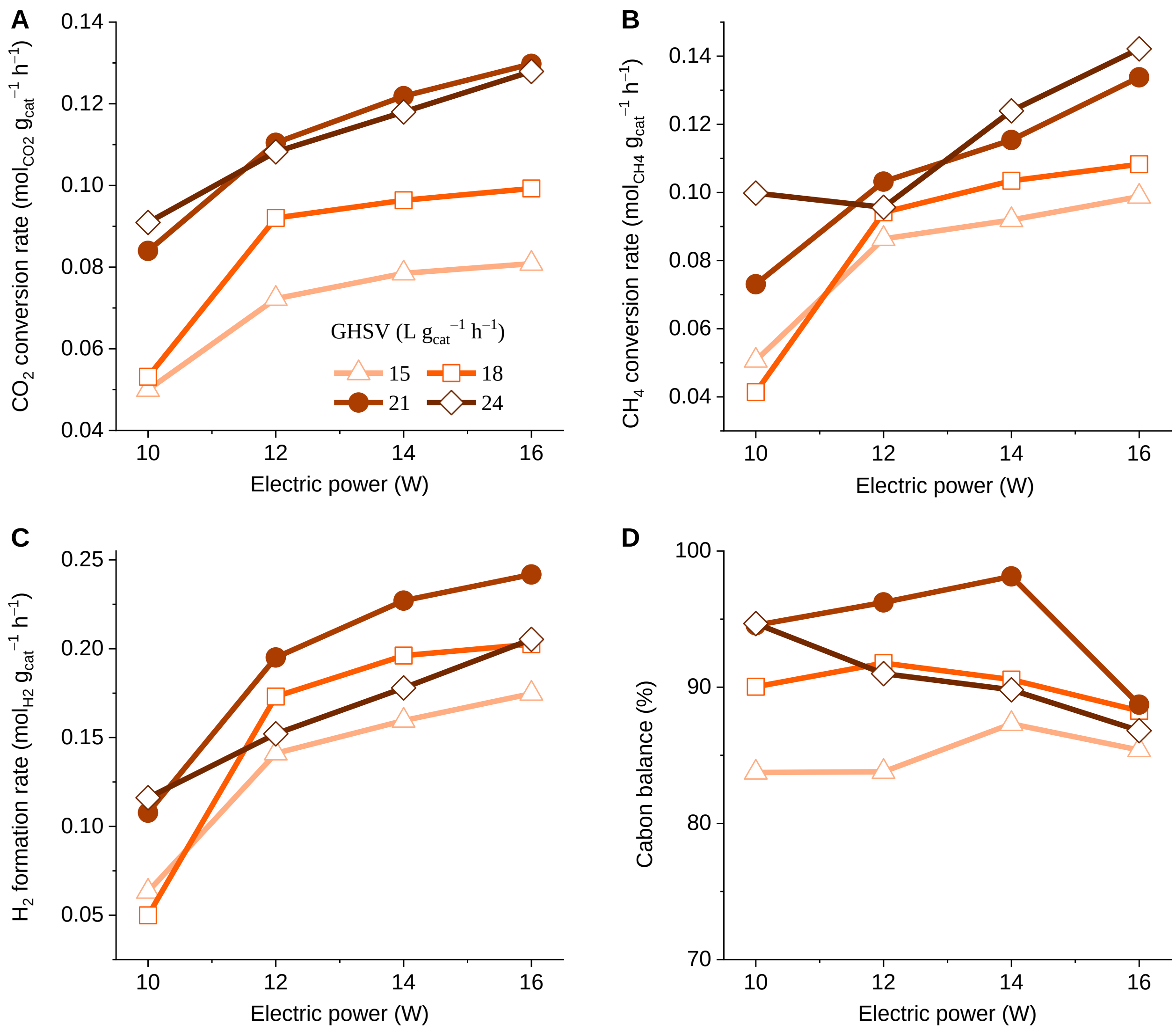

Fig. S5. Optimization of GHSV for e-DRM over Ni-La_2O_3/AC. (**A**) CO_2 conversion rate as a function of electric power. (**B**) CH_4 conversion as a function of electric power. (**C**) H_2 formation rate as a function of electric power. (**D**) Carbon balance as a function of electric power. The reactions were conducted with a CH_4/CO_2 ratio of 2, and the Ni-La_2O_3/AC catalyst has a Ni content of 4 wt.% and a La/Ni atomic ratio of 2.

To maximize the reaction rate of DRM, the GHSV was adjusted within 15–24 L h^{-1} g_{cat}^{-1} for the e-DRM over the optimized Ni-La_2O_3/AC catalysts. The CO_2 conversion rates are improved as the GHSV reaches up to 21 L h^{-1} g_{cat}^{-1}, and then do not obviously change when the GHSV approach to 24 L h^{-1} g_{cat}^{-1}, suggesting the reaction has reached to a steady-state. Therefore, the GHSV of 21 L h^{-1} g_{cat}^{-1} can considered as a critical point to minimize the external diffusion resistance for DRM. Beyond the critical GHSV, the formation rates of H_2 and as well as the carbon balance is decreased, which may be due to the high GHSV accelerates the carbon deposition from CO dissociation (*35, 36*).

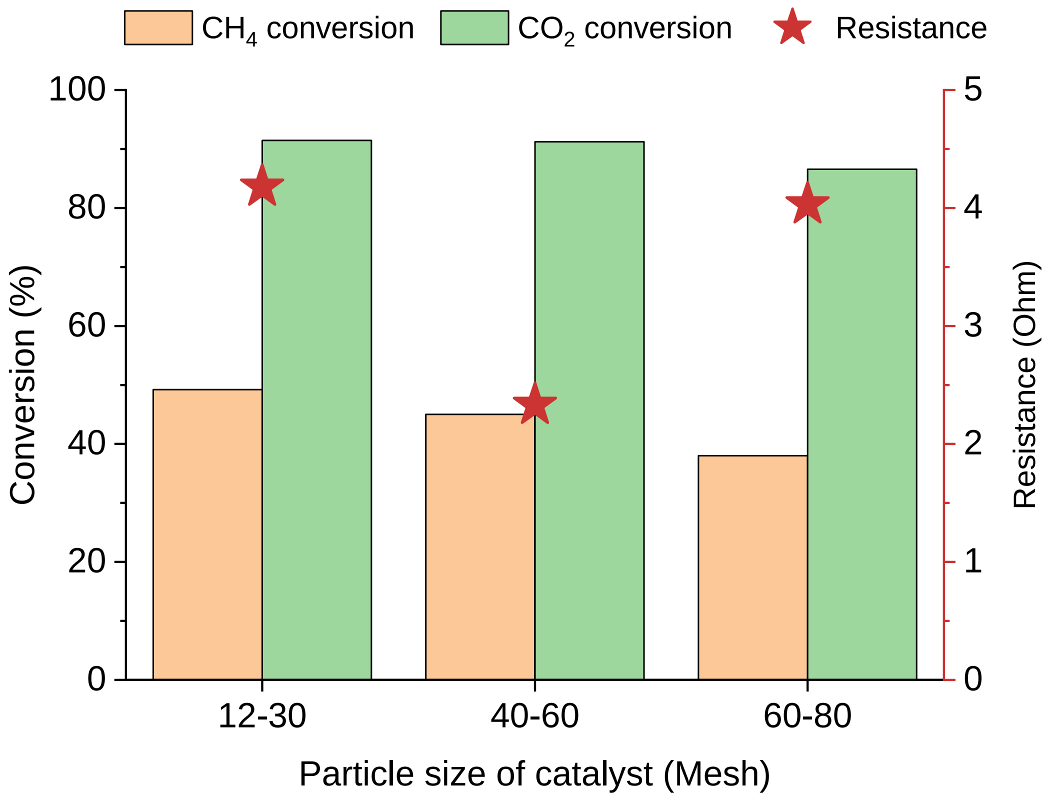

Fig. S6. e-DRM performance as functions of particle sizes of the Ni-La_2O_3/AC catalyst and the corresponding resistance of the catalyst bed. The Ni-La_2O_3/AC catalyst has a Ni content of 4 wt.% and a La/Ni atomic ratio of 2. The input electric power is 16 W.

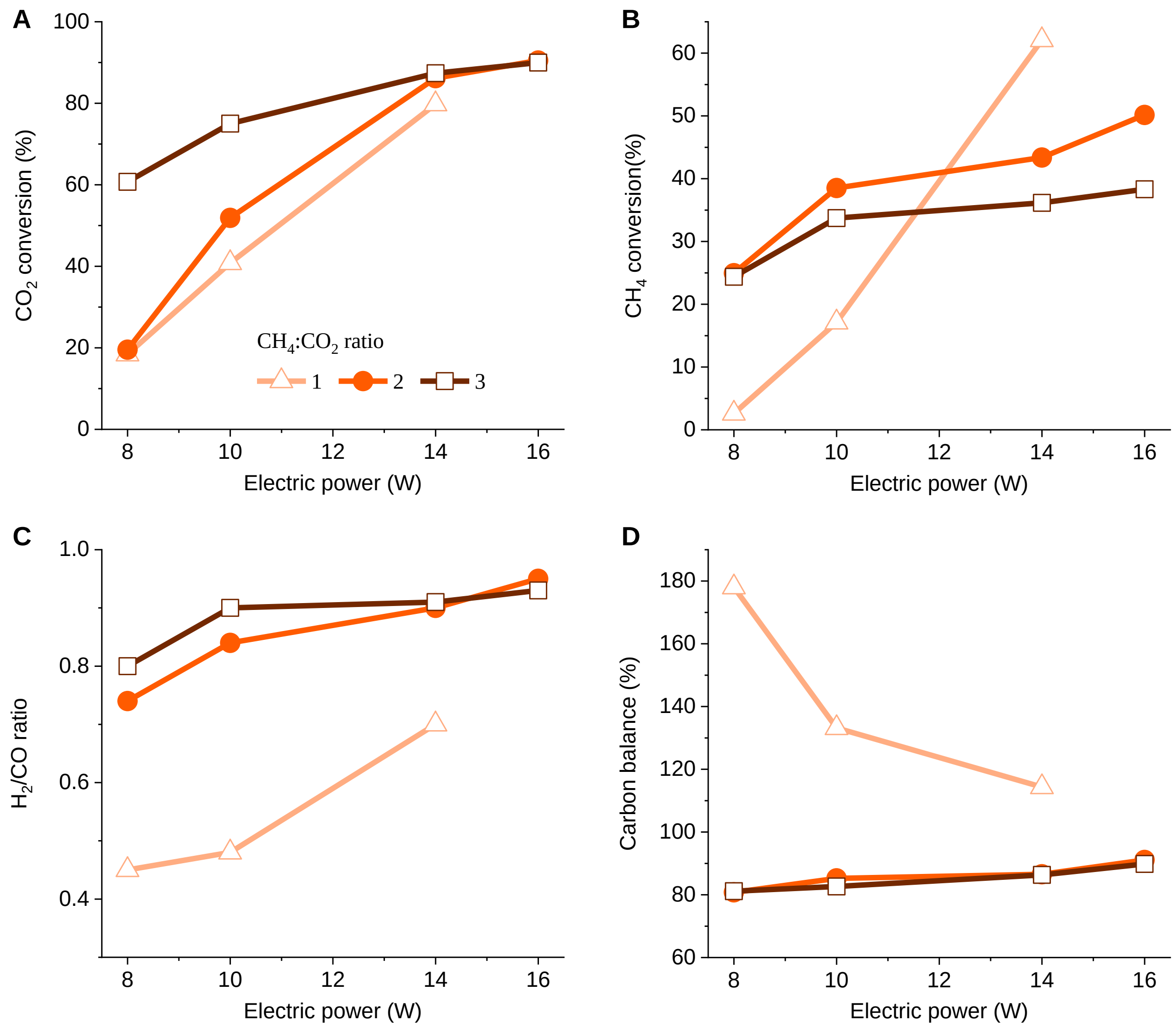

Fig. S7. Optimization of CH_4/CO_2 ratios for e-DRM over Ni-La_2O_3/AC. (**A**) CO_2 conversion as a function of electric power. (**B**) CH_4 conversion as a function of electric power. (**C**) H_2/CO ratio as a function of electric power. (**D**) Carbon balance as a function of electric power. The reactions were conducted with a GHSV of 21 L h^{-1} g_{cat}^{-1} and the Ni-La_2O_3/AC catalyst has a Ni content of 4 wt.% and a La/Ni atomic ratio of 2.

Three CH_4/CO_2 ratios, 1, 2 and 3, were selected for the e-DRM over a Ni-La_2O_3/AC catalyst. When the CH_4/CO_2 ratio increases from 1 to 3, the CO_2 conversion and the H_2/CO ratio are improved. When the CH_4/CO_2 ratio is unity, that the carbon balance always exceeds 100%, albeit with a decrease as the power increase, starting from 178% at 8 W of power. The excessive carbon balance originates from carbon gasification of the AC supports by CO_2 ($C + CO_2 \rightarrow 2CO$) (*37-39*). When the power is elevated to 16 W, the accumulated depletion of the AC support becomes so severe that the catalyst bed breaks, opening the circuit and discontinuing the electrified reaction. When the CH_4/CO_2 ratio increases to 2 and 3, the carbon balance gradually increase as the power is elevated but no longer exceed 100%, indicating that the DRM reaction completely overwhelms the gasification of AC. For the both CH_4/CO_2 ratios, the CO_2 conversion approaches to 100% with an approximate 0.9 of H_2/CO ratio when the electric power is above 14 W.

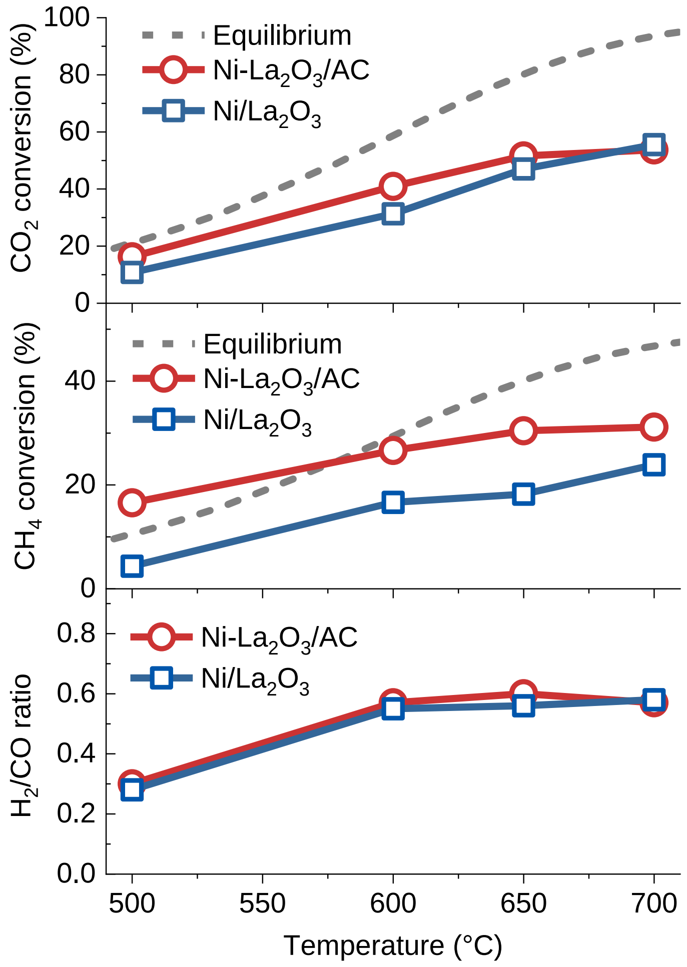

Fig. S8. Comparison in t-DRM performance between the Ni-La_2O_3/AC and Ni/La_2O_3 catalysts. The Ni-La_2O_3/AC catalyst has a Ni content of 4 wt.% and a La/Ni atomic ratio of 2; while the Ni/La_2O_3 catalyst has a Ni content of 4 wt.%. All reactions were conducted with a CH_4/CO_2 ratio of 2 and a GHSV of 21 L h^{-1} g_{cat}^{-1}.

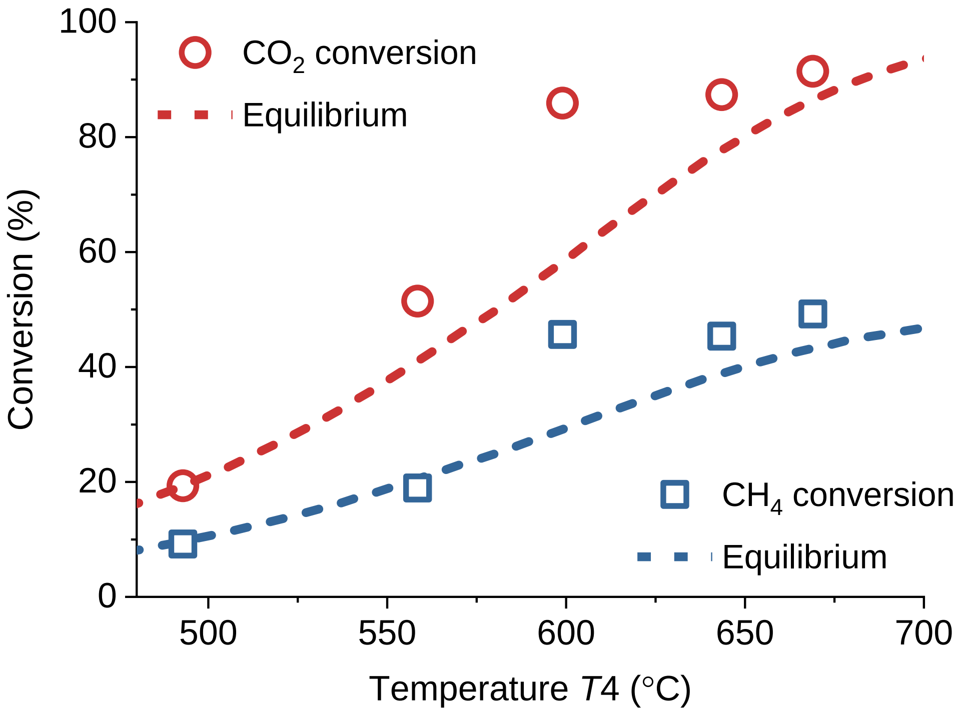

Fig. S9. Plots of CO_2 and CH_4 conversions for the e-DRM as functions of *T*4 temperature with thermodynamic equilibrium plots of DRM. The reactions were conducted with a GHSV of 21 L h^{-1} g_{cat}^{-1} and the Ni-La_2O_3/AC catalyst has a Ni content of 4 wt.% and a La/Ni atomic ratio of 2.

The CO_2 and CH_4 conversions for the e-DRM are plotted as functions of *T*4 temperature, which corresponds to the measurement location near the catalyst bed exit (Fig. 1A). Most data points do not notably deviate from the thermodynamic equilibrium plots, suggesting the majority of the catalyst bed operates within the thermodynamic equilibrium of DRM.

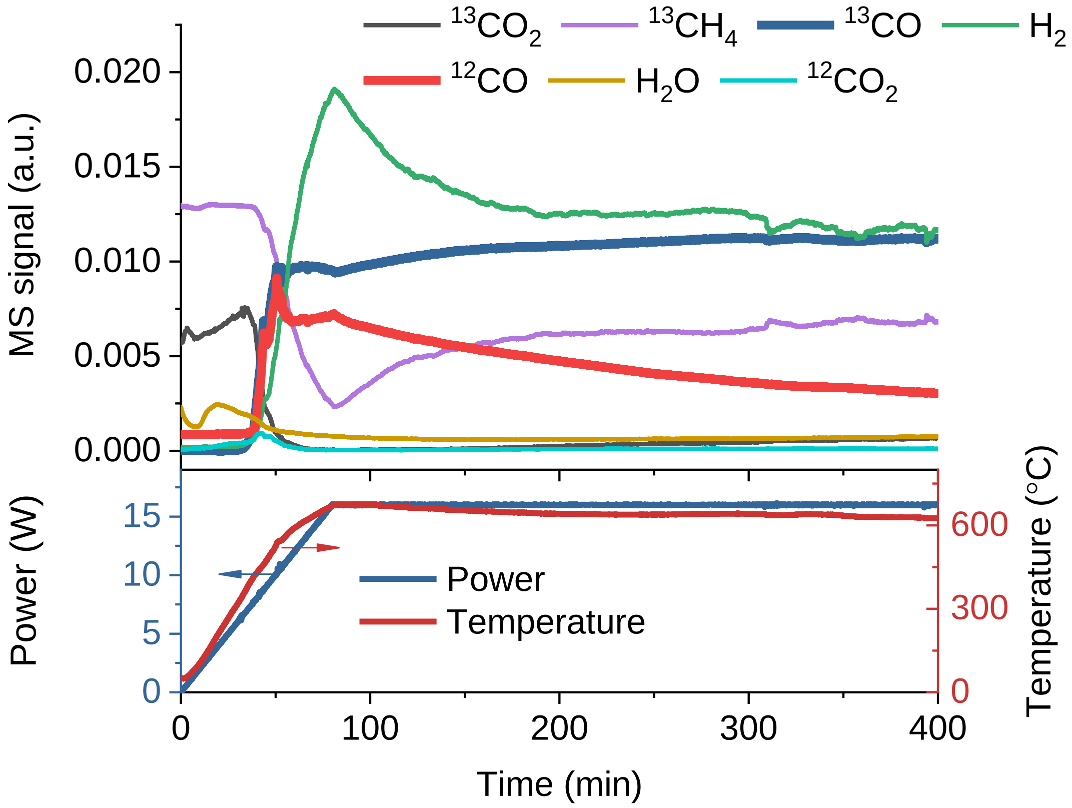

Fig. S10. Isotopic e-DRM reaction over Ni-La_2O_3/AC using $^{13}CH_4$ (0.67 vol.%) and $^{13}CO_2$ (0.33 vol.%) feeds. The Ni-La_2O_3/AC catalyst has a Ni content of 4 wt.% and a La/Ni atomic ratio of 2. The electric power is increased from 0 to 16 W power at rate of 0.2 W min^{-1} and then kept at 16 W.

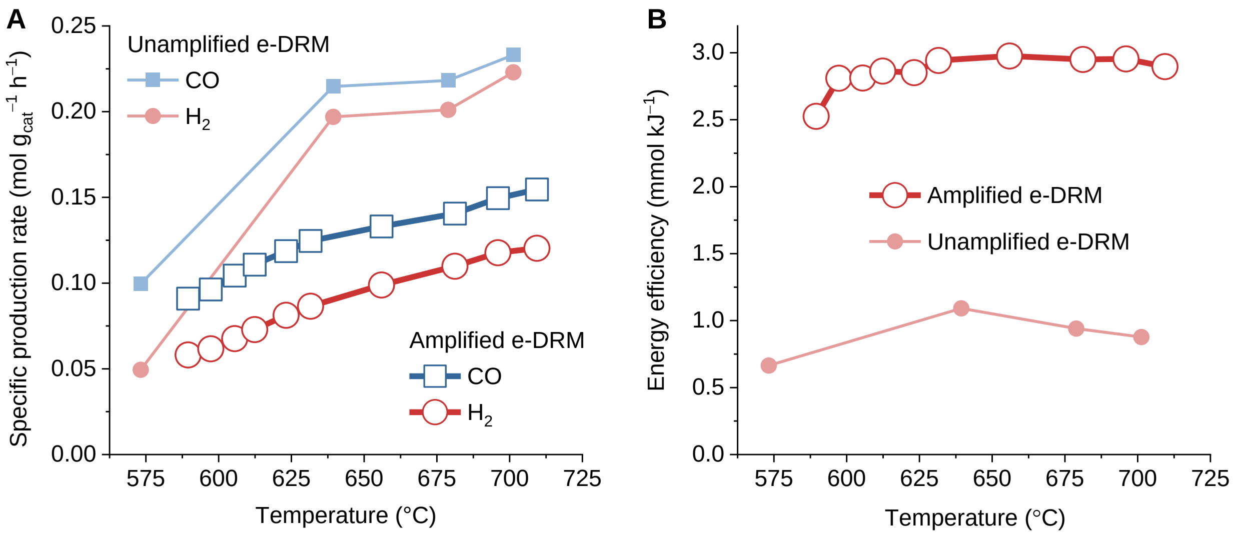

Fig. S11. Performances of the amplified e-DRM over Ni-La_2O_3/AC in comparison to that of the unamplified e-DRM. (**A**) Specific production rates of CO and H_2 as functions of temperatures. (**B**) Energy efficiency as functions of temperatures. The amplified reaction was conducted with a catalyst mass of 5.4 g and a CH_4/CO_2 ratio of 2. The GHSVs for CH_4 and CO_2 flows are 5.6 and 2.8 L h^{-1} g_{cat}^{-1}, respectively. The Ni-La_2O_3/AC catalyst has a Ni content of 4 wt.% and a La/Ni atomic ratio of 2.

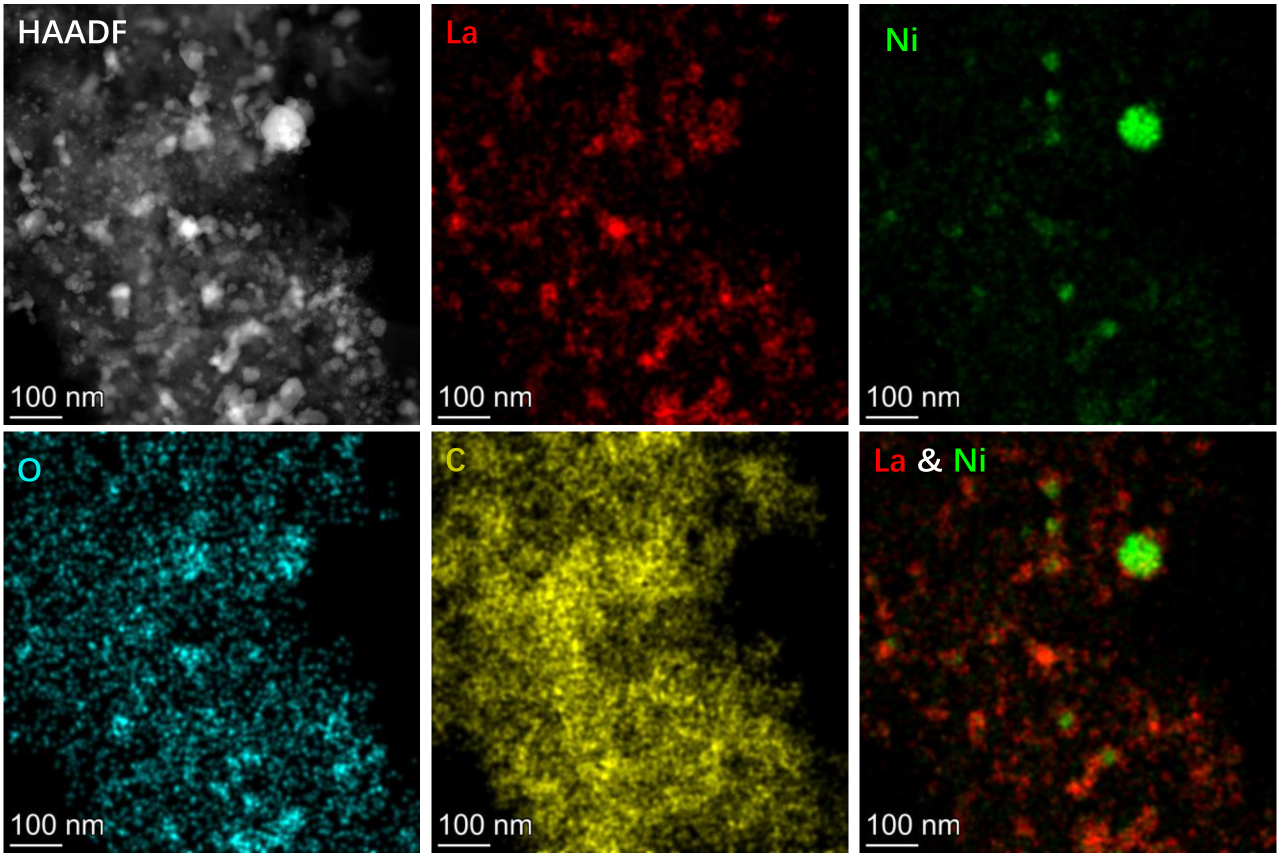

Fig. S12. HAADF-STEM image and accompanying EDX mapping for the fresh Ni-La_2O_3/AC catalyst. The Ni content is 4 wt.% and the La/Ni atomic ratio is 2.

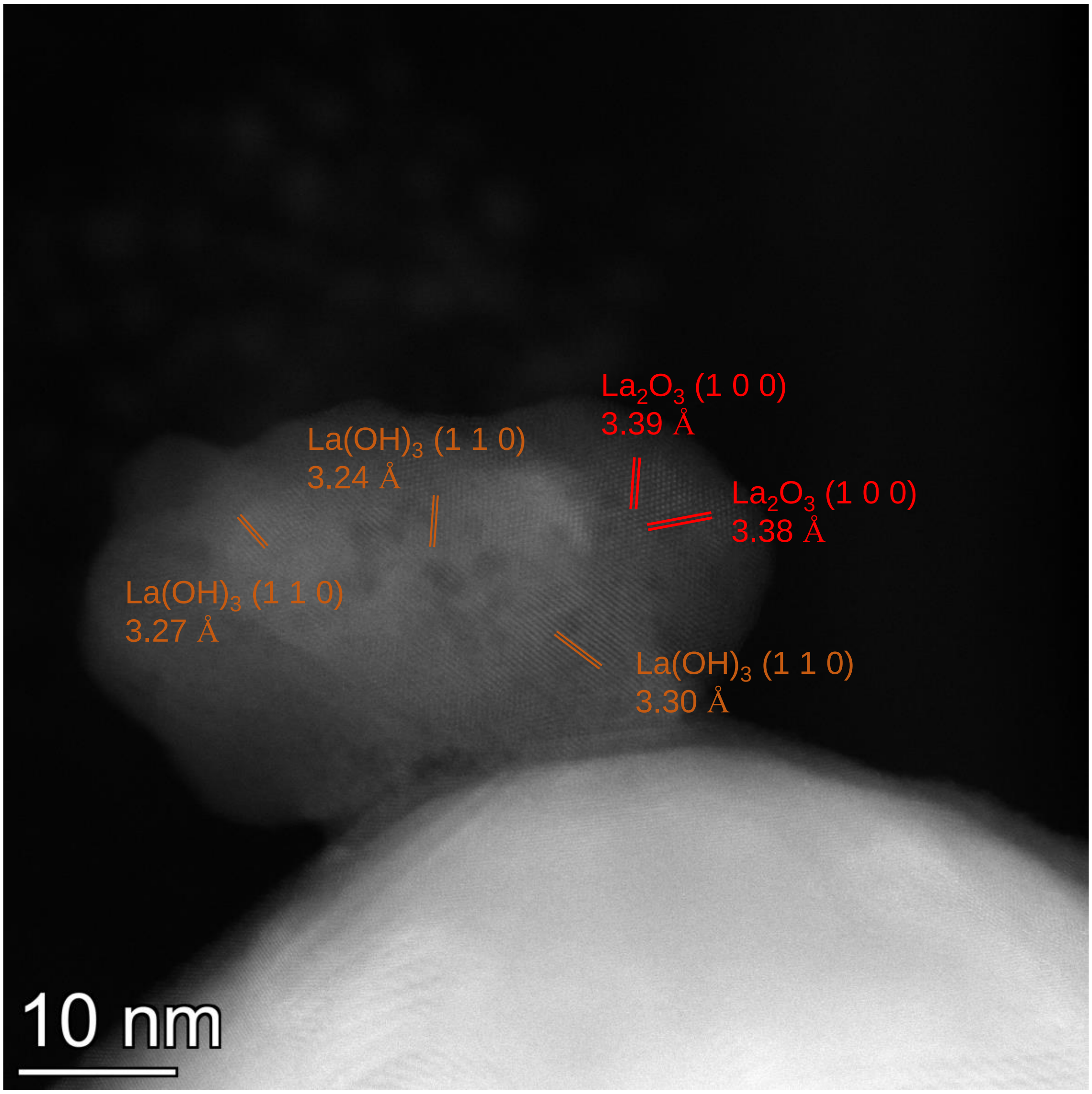

Fig. S13. HAADF-STEM image and lattice analysis for the Ni-La_2O_3/AC catalyst spent after the e-DRM. The lattice analysis is referring to the standard diffractions La_2O_3 (JCPDS 00-005-0602) and $La(OH)_3$ (JCPDS 01-083-2034). The Ni content is 4 wt.% and the La/Ni atomic ratio is 2. The e-DRM reaction was conducted at 16 W for 10 h, reaching around 700 °C, with a GHSV of 21 L h^{-1} g_{cat}^{-1} and a CH_4/CO_2 ratio of 2.

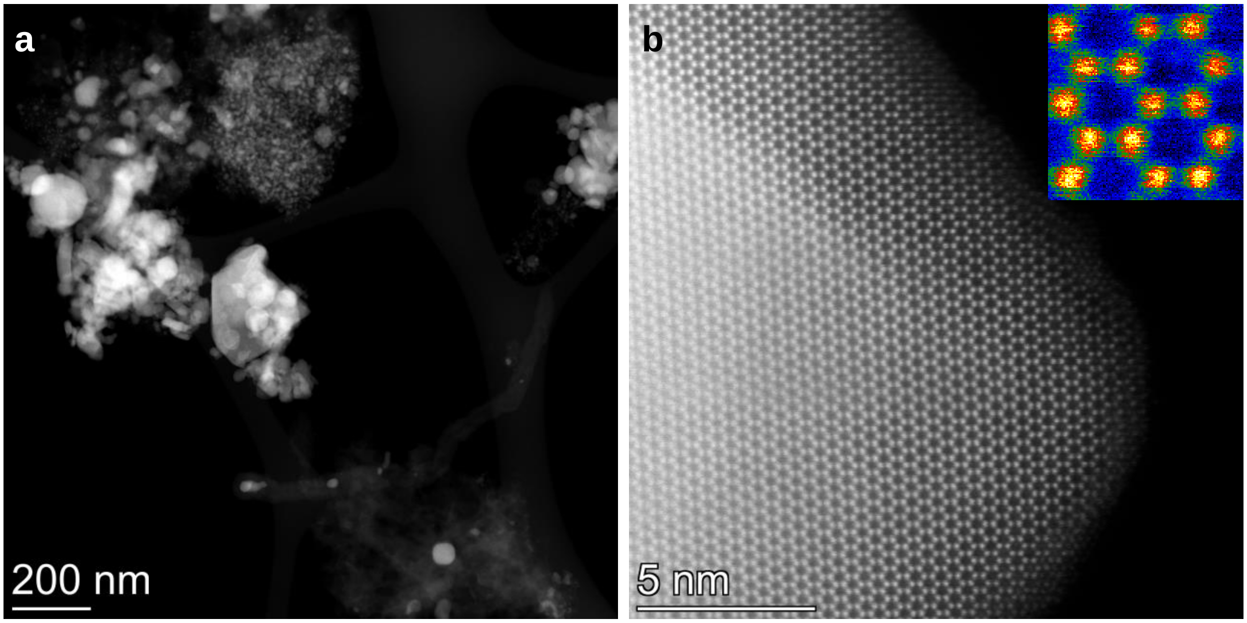

Fig. S14. HAADF-STEM image of the Ni-La_2O_3/AC catalyst spent after the e-DRM (a) and magnified image of the $La_2O_2CO_3$ species therein (b). The Ni content is 4 wt.% and the La/Ni atomic ratio is 2. The insert in the subfigure of b clearly shows the distinct, intense spots, indicative of the La atoms within the hexagonal lattice of $La_2O_2CO_3$. The e-DRM reaction was conducted at 16 W for 10 h, reaching around 700 °C, with a GHSV of 21 L h^{-1} g_{cat}^{-1} and a CH_4/CO_2 ratio of 2.

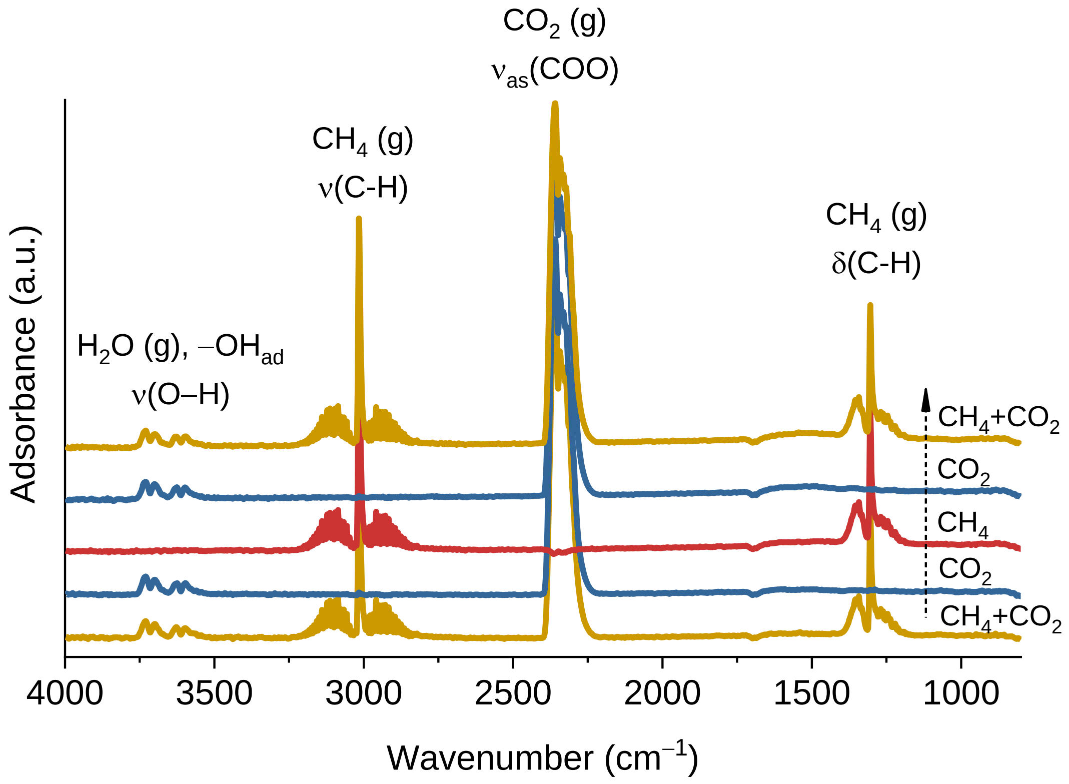

Fig. S15. Full spectra of *in situ* DRIFTS for the t-DRM over Ni-La_2O_3/AC at 500 °C during gas switching. The Ni content is 4 wt.% and the La/Ni atomic ratio is 2.

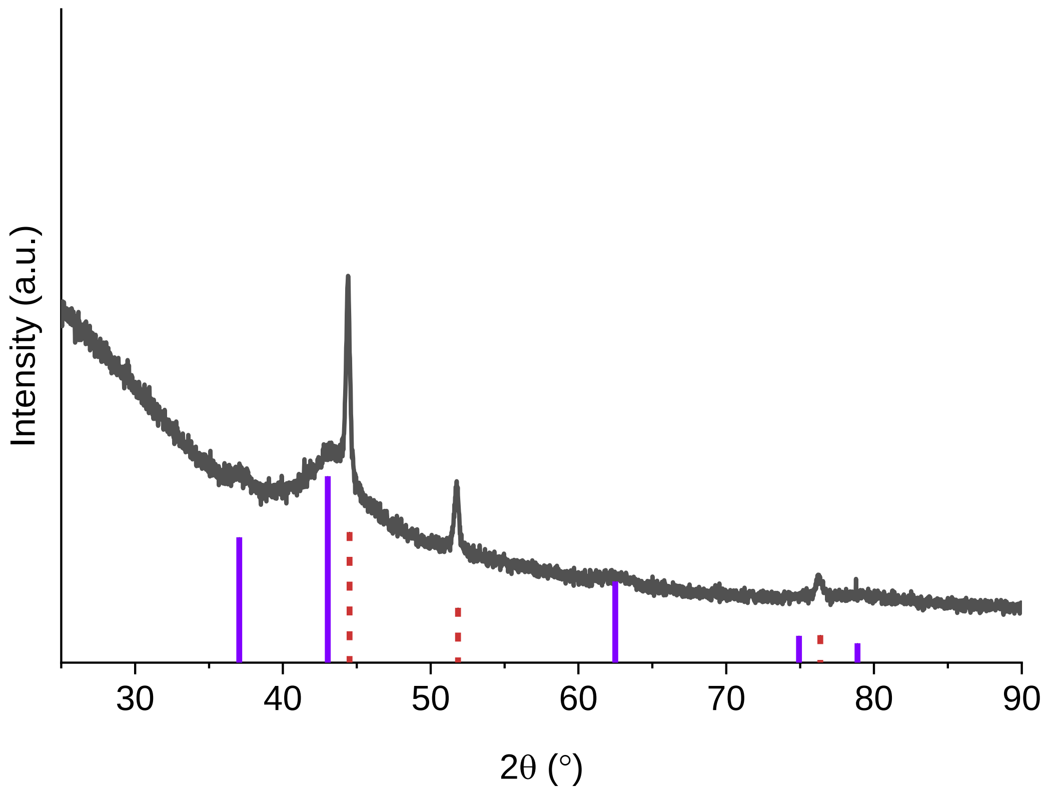

Fig. S16. XRD pattern of the Ni/AC catalyst after the oxidation by O_2 at 300 °C. The standard diffractions of NiO (JCPDS 01-071-4750 in purple solid line) and Ni (JCPDS 00-004-0850 in red dashed line) are referred. The Ni/AC has 4 wt.% of Ni content.

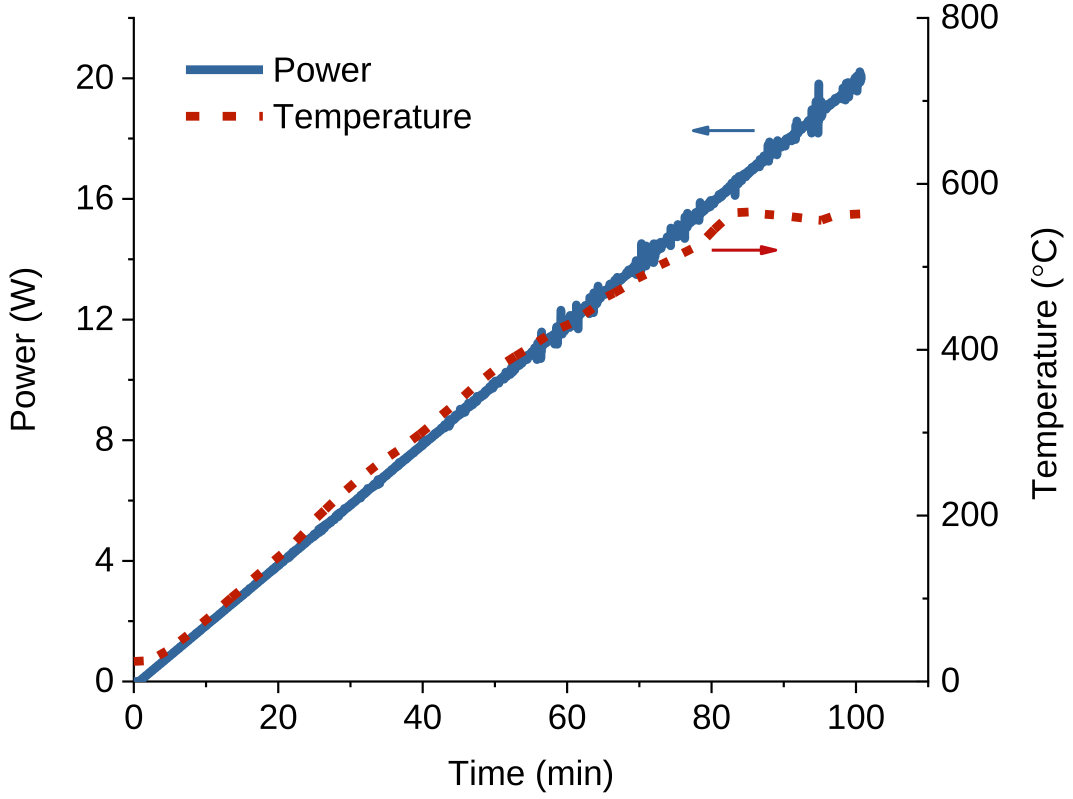

Fig. S17. Plots of electric power and temperature as functions of time in the EPPR for electrified NiO_x reduction. The test was carried out over the Ni/AC catalyst with 4 wt.% of Ni content, with a power ramp rate of 0.2 W min^{-1}. The NiO_x species was fabricated by treating the catalyst under an O_2 flow at 300 °C.

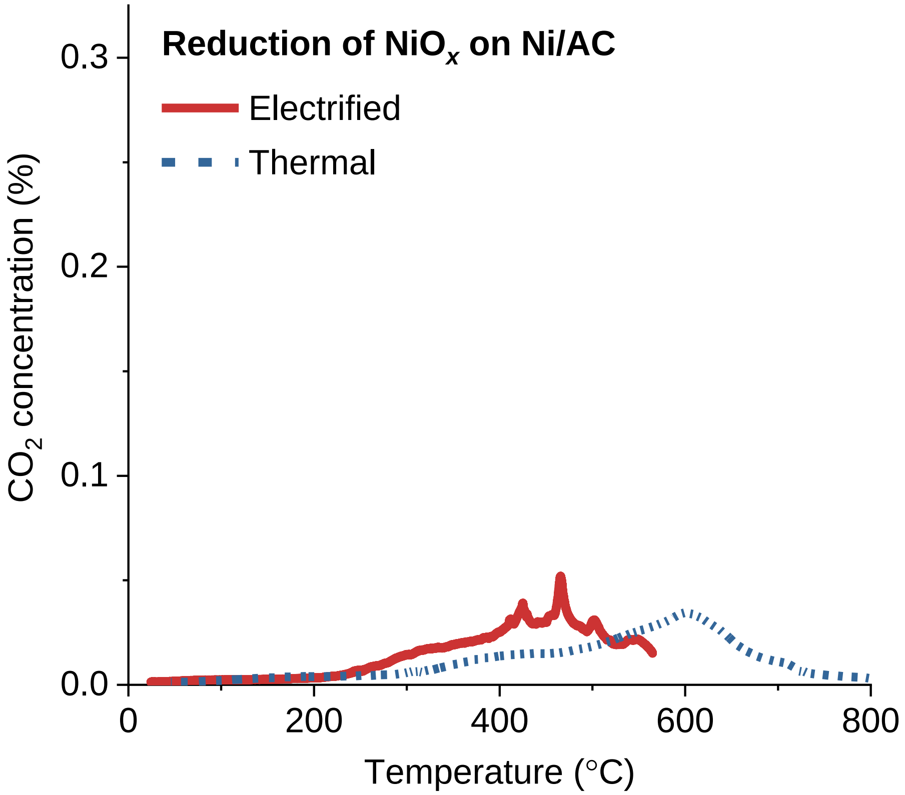

Fig. S18. CO_2 concentration as a function of temperature for the reduction of NiO_x on the Ni/AC catalyst in both the electrified EPPR and the thermal TPR. The catalyst contains 4 wt.% of Ni, and the NiO_x species was fabricated by treating the Ni/AC catalyst under an O_2 flow at 300 °C. The electrified EPPR was performed under an Ar flow at a power ramp rate of 0.2 W min^{-1}, while the thermal TPR was conducted under an Ar flow at at a heating rate of 10 °C min^{-1}.

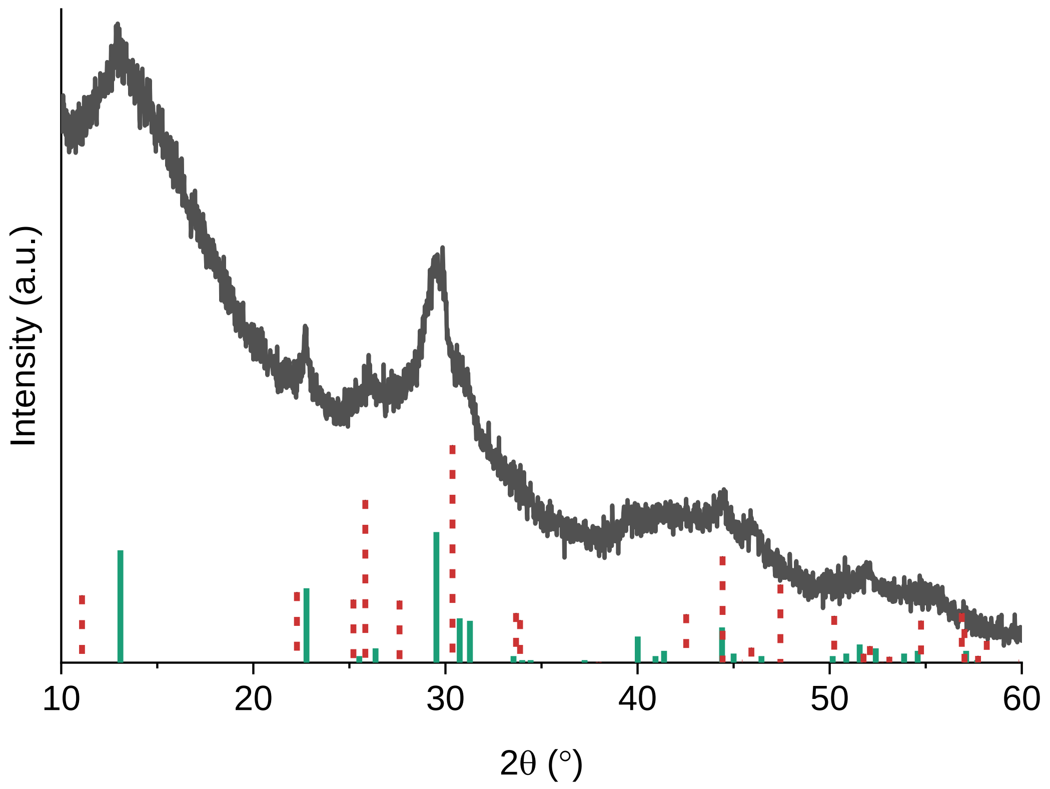

Fig. S19. XRD pattern of the La_2O_3/AC catalyst after CO_2 adsorption at 500 °C. The standard diffractions of monoclinic $La_2O_2CO_3$ (JCPDS 00-048-1113 in green solid line) and hexagonal $La_2O_2CO_3$ (JCPDS 04-009-3944 in red dashed line) are referred. The La_2O_3 content is 22 wt.%.

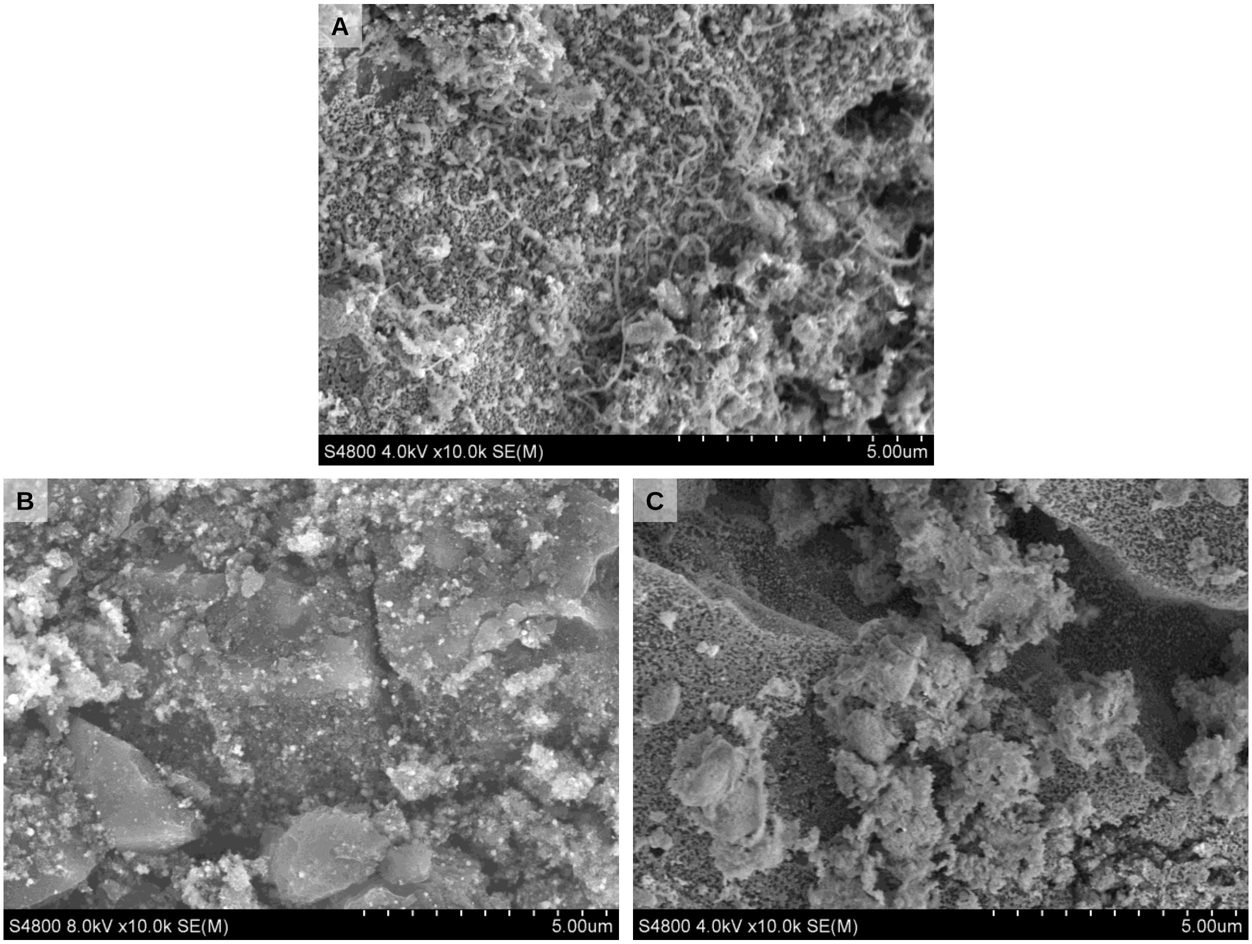

Fig. S20. Post SEM images of the Ni-La_2O_3/AC catalyst spend after the electrified CH_4 pyrolysis (A) and the e-DRM (B) and the t-DRM (C). The Ni content is 4 wt.% of and the La/Ni atomic ratio is 2. Both the e-DRM and t-DRM were conducted at 700 °C for 10 h with a GHSV of 21 L h^{-1} g_{cat}^{-1} and a CH_4/CO_2 ratio of 2.

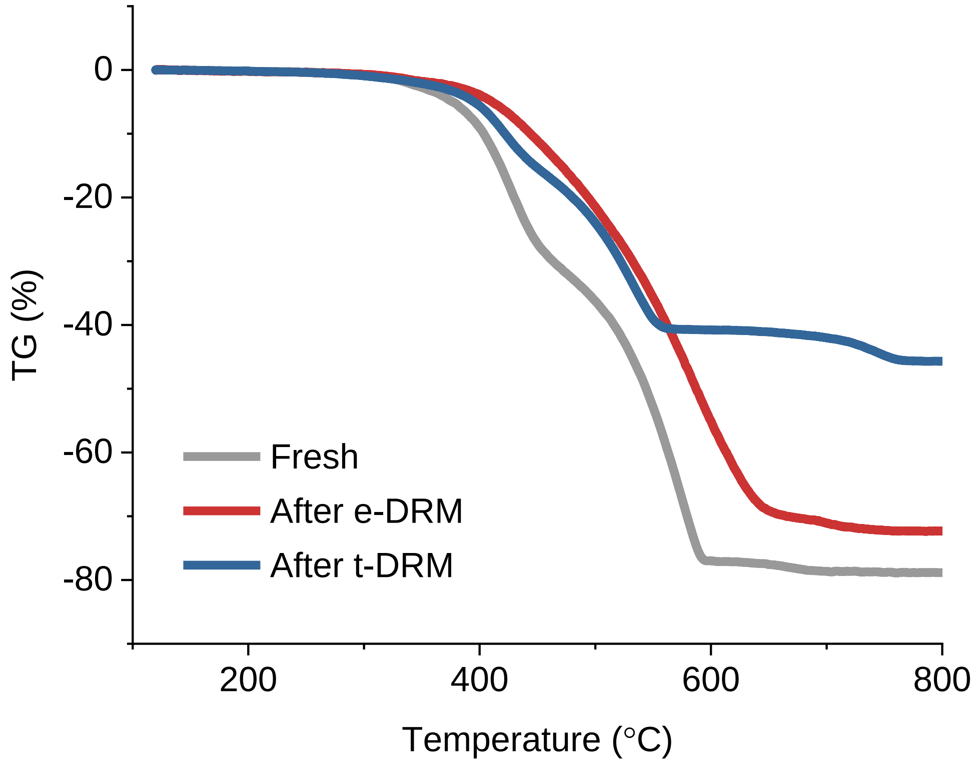

Fig. S21. TG curves for the fresh Ni-La_2O_3/AC catalyst and the two spent catalysts after the e-DRM and t-DRM reactions. The Ni content is 4 wt.% of and the La/Ni atomic ratio is 2. Both the DRM reactions were conducted at 700 °C for 10 h with a GHSV of 21 L h^{-1} g_{cat}^{-1} and a CH_4/CO_2 ratio of 2.

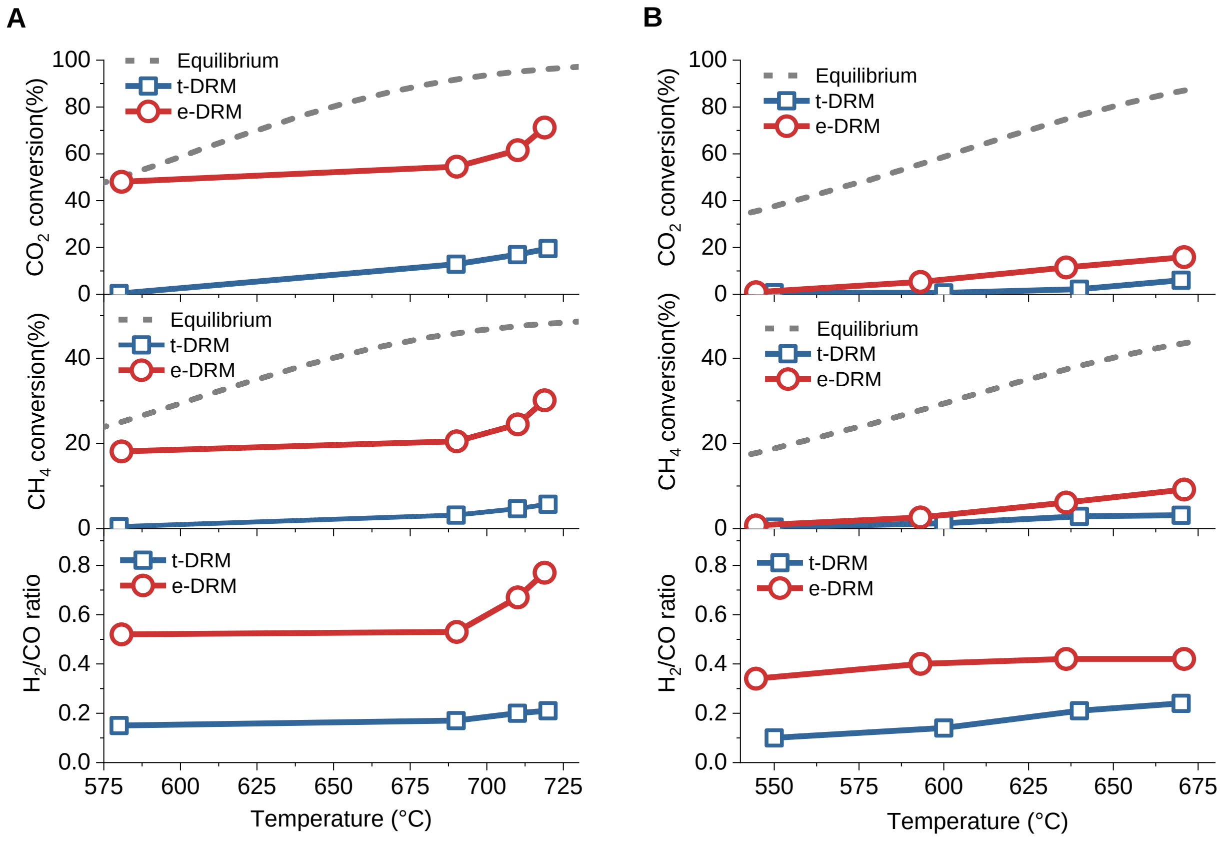

Fig. S22. Performance comparison between e-DRM and t-DRM over the Ni-CeO_2/AC (A) and the Ni-MgO/AC (B) catalysts. The Ni-CeO_2/AC catalyst has a Ni content of 4 wt.% and a Ce/Ni atomic ratio of 2; while the Ni-MgO/AC catalyst has a Ni content of 4 wt.% and a Mg/Ni atomic ratio of 2. All reactions were conducted with a CH_4/CO_2 ratio of 2 and a GHSV of 21 L h^{-1} g_{cat}^{-1}.

Supplementary Notes

Note S1. Calculation method of dimensionless energy efficiency for e-DRM

The input and output enthalpy flows were evaluated under the assumptions of ideal mixture and perfect gas according to the following relation (*20*):

$$\dot{H} = \sum \dot{n}_i \cdot h_i(T) = \sum \dot{n}_i \cdot (\Delta h^{\circ}_{F,i}\left(T_{ref}\right) + \int_{T_{ref}}^{T} C_{P,i}(T)\, dt) \quad \text{(Equation S1)}$$

where $\dot{n}_i$ is the molar flow rate, $h_i(T)$ is the enthalpy, T_{ref} is the reference temperature (298.15 K), $\Delta h^{\circ}_{F,i}(T_{ref})$ is the enthalpy of formation at T_{ref} and $C_{P,i}$is the specific heat capacity. These thermodynamic constants of reactants and products are listed in table S3. In this regard, the reaction heat duty (Q) was calculated according to the following equation:

$$Q = \dot{H}_{out} - \dot{H}_{in} \quad \text{(Equation S2)}$$

where $\dot{H}_{in}$ and $\dot{H}_{out}$ are the enthalpy flows of the gas mixtures at inlet and outlet of the reactor, respectively. In this regard, the power loss (P_{loss}) as well as the energy efficiency (η) can be calculated according to Eq. 3 and 4, respectively:

$$P_{loss} = P - Q \quad \text{(Equation S3)}$$

$$\eta = \mathrm{Q}/P \quad \text{(Equation S4)}$$

Note S2. Derivation of the formula for calculating the contribution of the MvK pathway to DRM reactions

The mechanistic pathway of the DRM reactions over the Ni-La_2O_3/AC catalyst involving $C^{18}O_2$ and CH_4 is simplified by the following reactions.

CH_4 dissociation:

$CH_4 + Ni_s \rightarrow C\text{-}Ni_s + 2H_2$ (Reaction 1)

L-H pathway:

$C^{18}O_2 + LO_s \rightarrow LCO_s$ (Reaction 2)

$LCO_s(^{18}O_2^*) + C\text{-}Ni_s \rightarrow 2C^{18}O + Ni_s + LO_s$ (Reaction 3)

MvK pathway:

$C^{18}O_2 + LO_{bulk} \rightarrow LCO_{bulk}$ (Reaction 4)

$LCO_{bulk}(^{18}O_2^*) + C\text{-}Ni_s \rightarrow 2C^{18}O + Ni_s + LO_{bulk}$ (Reaction 5)

$LCO_{bulk}(^{18}O^{16}O^*) + C\text{-}Ni_s \rightarrow C^{18}O + C^{16}O + Ni_s + LO_{bulk}$ (Reaction 6)

where, Ni_s denotes surface Ni; LO_s and LO_{bulk} represent the surface and bulk of La_2O_3 in the catalyst, respectively; LCO_s and LCO_{bulk} represent the surface and bulk $La_2O_2CO_3$ intermediates, which originates from CO_2 chemisorption on the surface of La_2O_3 (leading to LCO_s through Reaction 2) and within the bulk of La_2O_3 (leading to LCO_{bulk} through Reaction 4), respectively.

In the L-H pathway, the active oxygens of the surface $La_2O_2CO_3$ are two ^{18}O isotopes from $C^{18}O_2$, which is specifically denoted as $LCO_s(^{18}O_2^*)$. Since the catalyst's native oxygen (^{16}O) does not participate in the L-H pathway, all the active oxygens originate from $^{18}CO_2$.

In the MvK pathway, the catalyst's native oxygen (^{16}O) participates in the formation of bulk $La_2O_2CO_3$ from CO_2 chemisorption. Thereby, two types of bulk $La_2O_2CO_3$ are considered. One contains two active ^{18}O isotopes, denoted as $LCO_{bulk}(^{18}O_2^*)$; whereas the other contains one active ^{18}O and one active ^{16}O isotope, denoted as $LCO_{bulk}(^{18}O^{16}O^*)$.

The rates of these reactions are represented by R_i, where i is the reaction number.

The total rate of CO generation can be calculated by

$R_{\text{Total}} = 2R_3 + 2R_5 + 2R_6$ (Equation S5)

The CO generation rate via L-H pathway can be calculated by

$R_{\text{L-H}} = 2R_3$ (Equation S6)

The CO generation rate via MvK pathway can be calculated by

$R_{\text{MvK}} = 2R_5 + 2R_6$ (Equation S7)

The contribution of MvK pathway to CO generation (μ_{Mvk}) can be calculated by

$$\mu_{\mathrm{MvK}} = \frac{R_{\mathrm{MvK}}}{R_{\mathrm{Total}}} \times 100\% = \frac{2R_5+2R_6}{R_{\mathrm{Total}}} \times 100\% \quad \text{(Equation S8)}$$

Given the fact that the CO generation rates are proportional to the concentrations of the outlet CO, we obtain:

$$R_{\mathrm{Total}} = k_{\mathrm{g}}([\mathrm{C^{18}O}]_{\mathrm{s}} + [\mathrm{C^{16}O}]_{\mathrm{s}}) \quad \text{(Equation S9)}$$

$$R_6 = k_{\mathrm{g}}([\mathrm{C^{16}O}]_{\mathrm{s}}) \quad \text{(Equation S10)}$$

$$\frac{R_6}{R_{\mathrm{Total}}} = \frac{[\mathrm{C^{16}O}]_{\mathrm{s}}}{[\mathrm{C^{18}O}]_{\mathrm{s}}+[\mathrm{C^{16}O}]_{\mathrm{s}}} \quad \text{(Equation S11)}$$

where $[C^{18}O]_s$ and $[C^{16}O]_s$ represent the concentrations of outlet $C^{18}O$ and $C^{16}O$, respectively; k_g is the corresponding conversion constant. These concentrations are selected from the data points collected at the instant when the feed of $C^{18}O_2$ is about to stopped in Fig. 6B–C in the main text, which are close to steady-state conditions.

According to the law of mass action for Reactions 5 and 6, we obtain:

$$\frac{R_5}{R_6} = \frac{[\mathrm{LCO_{bulk}}(^{18}\mathrm{O_2}^*)]_{\mathrm{s}}}{[\mathrm{LCO_{bulk}}(^{18}\mathrm{O}^{16}\mathrm{O}^*)]_{\mathrm{s}}} \quad \text{(Equation S12)}$$

where $[LCO_s(^{18}O_2^*)]_s$ and $[LCO_{bulk}(^{18}O^{16}O^*)]_s$ represent the concentrations of $LCO_s(^{18}O_2^*)$ and $LCO_{bulk}(^{18}O^{16}O^*)$, respectively, which also correspond to the data points collected at the instant when the feed of $C^{18}O_2$ is about to stopped.

By substituting Equations S11–S12 into Equation S8, we obtain:

$$\mu_{\mathrm{MvK}} = 2 \times \left(\frac{[\mathrm{LCO_{bulk}}(^{18}\mathrm{O_2}^*)]_{\mathrm{s}}}{[\mathrm{LCO_{bulk}}(^{18}\mathrm{O}^{16}\mathrm{O}^*)]_{\mathrm{s}}} + 1\right) \times \frac{[\mathrm{C^{16}O}]_{\mathrm{s}}}{[\mathrm{C^{16}O}]_{\mathrm{s}}+[\mathrm{C^{18}O}]_{\mathrm{s}}} \times 100\% \quad \text{(Equation S13)}$$

$[LCO_{bulk}(^{18}O^{16}O^*)]_s$ can be calculated based on the amounts of $C^{16}O$ generated from the reduction of $LCO_{bulk}(^{18}O^{16}O^*)$ after the cessation of $C^{18}O_2$ feed. According to Reaction 6, we obtain the following the integral formulas:

$$[\mathrm{LCO_{bulk}}(^{18}\mathrm{O}^{16}\mathrm{O}^*)]_{\mathrm{s}} = k_{\mathrm{S}} \int_0^t [\mathrm{C^{16}O}]_t dt \quad \text{(Equation S14)}$$

where t demotes the time elapsed after the cessation of $C^{18}O_2$ feed, as depicted in Fig. 6B–C; $[C^{16}O]_t$ represents the concentrations of outlet $C^{16}O$ at time t following the cessation of $C^{18}O_2$ feed; k_s is the corresponding conversion constant.

Both the reductions of $LCO_{bulk}(^{18}O_2^*)$ and $LCO_{bulk}(^{18}O^{16}O^*)$ contribute to the generation of $C^{18}O$ after the cessation of the $C^{18}O_2$ feed (Reactions 5 and 6), in which the amount of $C^{18}O$ from the reduction of $LCO_{bulk}(^{18}O^{16}O^*)$ is equal to that of $C^{16}O$ according to Reaction 6, while this amount can be subtracted from the total generation amount of $C^{18}O$ in the calculation of $LCO_{bulk}(^{18}O_2^*)$. Thereby, we obtain:

$$[\mathrm{LCO_{bulk}}(^{18}\mathrm{O_2}^*)]_{\mathrm{s}} = \left(k_{\mathrm{S}} \int_0^t [\mathrm{C^{18}O}]_t dt - k_{\mathrm{S}} \int_0^t [\mathrm{C^{16}O}]_t dt\right) \times \frac{1}{2} \quad \text{(Equation S15)}$$

where $[C^{18}O]_t$ represents the concentrations of outlet $C^{18}O$ at time t following the cessation of $C^{18}O_2$ feed;

By substituting Equation S14 and S15 into Equation S13, we obtain:

$$\mu_{\mathrm{MvK}} = \left(\frac{\int_0^t [\mathrm{C}^{18}\mathrm{O}]_t \mathrm{d}t}{\int_0^t [C^{16}O]_t dt} + 1\right) \times \frac{[\mathrm{C}^{16}\mathrm{O}]_\mathrm{s}}{[\mathrm{C}^{16}\mathrm{O}]_\mathrm{s} + [\mathrm{C}^{18}\mathrm{O}]_\mathrm{s}} \times 100\% \qquad \text{(Equation S16)}$$

Based on the data in Fig. 6B–C and Equation S16, μ_{MvK} values for the e-DRM and the t-DRM are calculated to be 16.1% and 29.5%, respectively.

Table S1. Reported energy efficiencies for DRM over different catalytic modalities.

Entry	Catalyst	Catalytic modality	Catalyst mass (g)	Power spent (W)	WHSV ($L\ h^{-1}\ g_{cat}^{-1}$)	CH_4/CO_2 ratio	Conversion (%)		Energy efficiency ($mmol\ kJ^{-1}$)	Ref.
							CH_4	CO_2		
1	Ni/Al_2O_3	Plasma	1.0	130	1.8	1/1	55.7	33.5	0.100	(*42*)
2	Ni/Al_2O_3	Plasma	0.5	30	6	1/1	26.1	16.3	0.110	(*43*)
3	Ni/Al_2O_3-MgO	Plasma	0.04	100	7.644	1/1	74.5	73	0.117	(*44*)
4	Ni-Fe/Al_2O_3	Plasma	0.2	160	12	1/1	68.65	60.5	0.120	(*45*)
5	Ni-$La_2O_3@SiO_2$	Plasma	-	160	15	1/1	66.8	56.9	0.144	(*46*)
6	$LaNiO_3@SiO_2$	Plasma	0.2	150	12	1/1	88.31	77.2	0.170	(*47*)
7	Pt/Al_2O_3	Plasma	11	45	0.273	1/1	32.5	22.1	0.205	(*48*)
8	Ni-Cu/Al_2O_3	Plasma	0.1	60	36	1/1	69	75	0.267	(*49*)
9	Ni/Al_2O_3	Plasma	1.0	50	3	1/1	56.4	30.2	0.322	(*50*)
10	Mo_2C-Ni/Al-2	Plasma	0.12	93.6	1750	1/1	16.2	20.0	0.640	(*51*)
11	Ni-K/Al_2O_3	Plasma	0.4	16	7.5	3/2	31.6	22.8	0.670	(*52*)
12	Mo_2C	Pulsed laser	0.02	16	30	1/1	50.2	−	0.980	(*41*)
13	Ni-La_2O_3/AC	e-DRM	0.2	12	21	2/1	45.67	85.9	1.091	This work
14	Ni-La_2O_3/AC	Amplified e-DRM	5.4	60	8.4 [a]	2/1	22.31	49.5	2.976	This work

[a] The amplified e-DRM maintained the GHSVs for CH_4 and CO_2 flows same to those of the unamplified e-DRM, which were 5.6 and 2.8 $L\ h^{-1}\ g_{cat}^{-1}$, respectively.

Table S2. Calculated net CO_2 emissions for H_2 production in the DRM reactions powered by electricity from different generation sources.

Electricity generation source	CO_2 emission factor (μ) (kg_{CO2} kWh^{-1}) [a]	Net CO_2 emission for H_2 production (C_{H2}) (kg_{CO2} kWh^{-1})		
		t-DRM	e-DRM	Amplified e-DRM
Offshore wind	0.009	8.25	−10.73	−13.98
Nuclear	0.0242	68.04	−8.39	−13.12
Hydropower	0.0749	267.45	−0.59	−10.28
Onshore wind	0.1237	459.4	6.91	−7.53
Solar thermal	0.15	562.84	10.96	−6.05
Photovoltaic	0.3	1152.83	34.03	2.38
Natural gas	0.499	1935.55	64.63	13.56
Oil	0.733	2855.93	100.62	26.71
Coal	0.888	3465.59	124.46	35.42

[a] The data is sourced from Ref. 37 (*Renewable Sustainable Energy Rev.* 39, 461-475 (2014)).

Table S3. Thermodynamic constants of reactants and products for DRM reactions

Gas	Composition	$\Delta h^{\circ}_{F,i}(T_{ref})$ [a] (kJ·mol^{-1})	$C_{P,i}(T)$ [a] (J·K^{-1}·mol^{-1})	T_{ref} [b] (K)	T [c] (K)
1	CH_4	-74.873	71.795	298.15	1000
2	CO_2	-393.522	54.308	298.15	1000
3	CO	-110.527	33.183	298.15	1000
4	H_2	0	30.205	298.15	1000

[a] The data is sourced from NIST-JANAF Thermochemical Tables (2021), https://janaf.nist.gov, accessed September 19, 2022.

[b] Ambient temperature.

[c] Reaction temperature.
